# Habitat differentiation among three Nigeria–Cameroon chimpanzee (*Pan troglodytes ellioti*) populations

**DOI:** 10.1002/ece3.4871

**Published:** 2019-01-10

**Authors:** Ekwoge E. Abwe, Bethan J. Morgan, Barthelemy Tchiengue, Fabrice Kentatchime, Roger Doudja, Marcel E. Ketchen, Eric Teguia, Ruffin Ambahe, Dana M. Venditti, Matthew W. Mitchell, Bernard Fosso, Albert Mounga, Roger C. Fotso, Mary Katherine Gonder

**Affiliations:** ^1^ Department of Biology Drexel University Philadelphia Pennsylvania; ^2^ Ebo Forest Research Project Yaoundé Cameroon; ^3^ San Diego Zoo Global San Diego California; ^4^ National Herbarium of Cameroon Yaoundé Cameroon; ^5^ Ministry of Forestry and Wildlife Yaoundé Cameroon; ^6^ Wildlife Conservation Society Yaoundé Cameroon; ^7^ Department of Biology University of Pennsylvania Philadelphia Pennsylvania

**Keywords:** ecological niche models, ecotone, ground‐truthing, human‐modified landscape, rainforest

## Abstract

Ecological niche models (ENMs) are often used to predict species distribution patterns from datasets that describe abiotic and biotic factors at coarse spatial scales. Ground‐truthing ENMs provide important information about how these factors relate to species‐specific requirements at a scale that is biologically relevant for the species. Chimpanzees are territorial and have a predominantly frugivorous diet. The spatial and temporal variation in fruit availability for different chimpanzee populations is thus crucial, but rarely depicted in ENMs. The genetic and geographic distinction within Nigeria–Cameroon chimpanzee (*Pan troglodytes ellioti*) populations represents a unique opportunity to understand fine scale species‐relevant ecological variation in relation to ENMs. In Cameroon, *P. t. ellioti *is composed of two genetically distinct populations that occupy different niches: rainforests in western Cameroon and forest–woodland–savanna mosaic (ecotone) in central Cameroon. We investigated habitat variation at three representative sites using chimpanzee‐relevant environmental variables, including fruit availability, to assess how these variables distinguish these niches from one another. Contrary to the assumption of most ENM studies that intact forest is essential for the survival of chimpanzees, we hypothesized that the ecotone and human‐modified habitats in Cameroon have sufficient resources to sustain large chimpanzee populations. Rainfall, and the diversity, density, and size of trees were higher at the rainforest. The ecotone had a higher density of terrestrial herbs and lianas. Fruit availability was higher at Ganga (ecotone) than at Bekob and Njuma. Seasonal variation in fruit availability was highest at Ganga, and periods of fruit scarcity were longer than at the rainforest sites. Introduced and secondary forest species linked with anthropogenic modification were common at Bekob, which reduced seasonality in fruit availability. Our findings highlight the value of incorporating fine scale species‐relevant ecological data to create more realistic models, which have implications for local conservation planning efforts.

## 1 INTRODUCTION

Ecological niche models (ENMs) are widely used to characterize habitat suitability for a species in a given location, and information from these models may be used to predict the species distribution patterns, densities, and trends (Junker et al., 2012; Sesink Clee et al., 2015). Several recent ENM studies estimate suitable habitats of apes using known ape distributions (Junker et al., 2012; Sesink Clee et al., 2015; Strindberg et al., 2018) and project ape population decline due to anthropogenic pressures and infectious disease risk, most notably resulting from Ebola; and climate change (Sesink Clee et al., 2015; Strindberg et al., 2018; Walsh et al., 2003). These studies rely upon global environmental datasets that describe several abiotic and biotic factors, such as tree cover, surface moisture, precipitation, and seasonality, generally sampled at 1‐km^2^ resolution (Dimiceli et al., 2011; Farr et al., 2007). However, it is often unclear how these variables directly relate to the resources available to species in their habitats, and most importantly, how the resources used by populations of apes correspond with these remotely sensed abiotic and biotic variables. Understanding these relationships is an important starting point in order to translate the relationships that exist between ENMs based on habitat suitability, and how these models apply at a scale that is ecologically relevant to ape communities and the resources that they rely on for survival.

**Figure 1:**
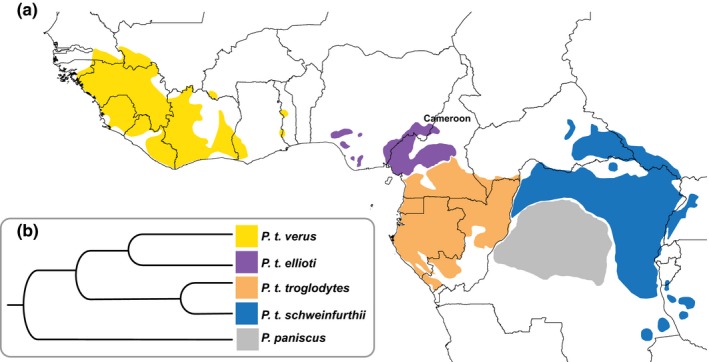
Distribution and phylogeny of the genus *Pan*. (a) The distribution of bonobos and chimpanzee subspecies. (b) Phylogenetic relationships of bonobos and chimpanzee subspecies inferred from complete genomes (Prado‐Martinez et al., 2013)

Habitats occupied by different chimpanzee populations vary (Stumpf, 2011) and could be important in understanding socioecological and genetic diversity in the species. Therefore, understanding the link between ENMs and chimpanzee‐specific habitat requirements is important. Four geographically distinct subspecies of chimpanzees occur in Africa, from Senegal in west Africa to Tanzania in the east (Figure 1a,b) (Caldecott & Miles, 2005). There is a western lineage that includes *Pan troglodytes *verus and *Pan troglodytes ellioti* and a central‐eastern lineage that includes *P. t. troglodytes* and *P. t. schweinfurthii* (Prado‐Martinez et al., 2013). The processes that have generated the distribution and diversity of these chimpanzee subspecies are largely unexplored (Mitchell & Gonder, 2013). Cameroon is of particular interest in this regard, as it constitutes an area of active chimpanzee diversification (Gonder et al., 2011; Gonder, Locatelli, Ghobrial, & Sheppard, 2009; Mitchell, Locatelli, Ghobrial, et al., 2015). The two main branches of the chimpanzee phylogenetic tree split at the Sanaga River in central Cameroon (Gonder, Disotell, & Oates, 2006; Gonder et al., 1997; Prado‐Martinez et al., 2013), and the river also marks the separation of *P. t. troglodytes* and *P. t. ellioti* (Gonder et al., 2011; Mitchell, Locatelli, Ghobrial, et al., 2015). There is a further population subdivision found within *P. t. ellioti*. There is one genetic population, or gene pool, associated with the mountainous rainforest habitats in western Cameroon, and a second genetic population found in the forest–woodland–savanna mosaic (ecotone) in central Cameroon (Mitchell, Locatelli, Sesink Clee, Thomassen, & Gonder, 2015). There is evidence that variation across these habitats plays an important role not only in sex‐specific community structuring (Mitchell, Locatelli, Abwe, Ghobrial, & Gonder, 2018), but also in the partitioning of genetic diversity within *P. t. ellioti* (Mitchell, Locatelli, Sesink Clee, et al., 2015).

Several studies have shown that habitat variation impacts many aspects of chimpanzee socioecology (Humle & Matsuzawa, 2001; Morgan & Abwe, 2006; Whiten et al., 1999; Yamakoshi, 1998). In general, chimpanzees are frugivores that live in fission–fusion communities of 20–150 individuals (Mitani, 2006; Sugiyama, 2004). They depend on the presence of standing trees to build nightly nests (Stanford & O'Malley, 2008) and fruiting trees for feeding through much of the year (Potts, Chapman, & Lwanga, 2009). Even within relatively uniform rainforest habitats, chimpanzee‐relevant resources are not evenly distributed, and in heterogeneous habitats, resources are highly clumped with considerable variation in availability through the year (Chapman, Chapman, Zanne, Poulsen, & Clark, ; Potts et al., 2009; White, 1994).

Fruit phenology varies seasonally and interannually within and between forest types (Anderson et al., 2005; Marshall et al., 2009; Potts et al., 2009). Seasonal variation in fruit availability and the quality of terrestrial herbaceous vegetation (THV) affect chimpanzee population and group dynamics (Tutin, Fernandez, Rogers, Williamson, & McGrew, 1991; Tutin, Ham, White, & Harrison, 1997; Wrangham et al., 1991). Chimpanzees in rainforest habitats have smaller home ranges (Herbinger, Boesch, & Rothe, 2001; Morgan & Sanz, 2006), relatively large foraging parties (Newton‐Fisher, 2003; Watts & Mitani, 2001), and a high dietary diversity in fleshy fruits (Deblauwe, 2009; Head, Boesch, Makaga, & Robbins, 2011; Morgan & Sanz, 2006; Watts, Potts, Lwanga, & Mitani, 2012). In drier and savanna habitats, chimpanzee home ranges are larger (Hunt & McGrew, 2002; McGrew, Baldwin, & Tutin, 1988; Pruetz & Bertolani, 2009), they have smaller foraging parties (McGrew et al., 1988; Ogawa, Idani, Moore, Pintea, & Hernandez‐Aguilar, 2007), and lower dietary diversity in fleshy fruits (Dutton & Chapman, 2015; Hunt & McGrew, 2002; McGrew et al., 1988).

Habitat differences are also reflected in chimpanzee nesting patterns with relatively larger parties associated with rainforest compared to drier and savanna habitats (Basabose & Yamagiwa, 2002; Brownlow, Plumptre, Reynolds, & Ward, 2001; Hunt & McGrew, 2002). However, in drier habitats where chimpanzees are also sympatric with predators, nesting parties are larger as smaller foraging parties congregate at nesting sites for safety (Ogawa et al., 2007). Ecological variation has also been linked with variation in grouping patterns between chimpanzee subspecies and bonobos: For example, eastern chimpanzee groups are male‐bonded (Wrangham & Smuts, 1980), western chimpanzees are bisexually‐bonded (Boesch, ), and bonobos are female‐bonded (Stanford, 1998).

A recent study that modeled habitat suitability for chimpanzees in Cameroon revealed that the two genetically distinctive *P. t. ellioti *subpopulations reported in Mitchell, Locatelli, Ghobrial, et al. (2015) occupy two significantly different niches that were significantly different from one another and from the niche occupied by *P. t. troglodytes* in southern Cameroon (Sesink Clee et al., 2015). Suitable habitats for *P. t. troglodytes* were fairly homogenous, especially in annual rainfall, forest cover and relief. In contrast, suitable habitats for *P. t. ellioti* were characterized by greater variation in precipitation and temperature seasonality, forest cover and relief (Sesink Clee et al., 2015). Differences in these environmental conditions were especially pronounced between the western mountainous rainforest and ecotone habitats (Sesink Clee et al., 2015), and the differences between them broadly corresponded with the distribution of the two genetically distinctive populations of *P. t. ellioti*.

However, while niche variation captured through ENMs is salient and informative, ecological details including forest structure, species richness, and fruit phenology that are important to frugivores cannot be depicted through such models. The chimpanzee range across Africa is marked by environmental and ecological variation, and differences in chimpanzees socioecology are tied to this variation, including feeding and nesting behaviors (Stumpf, 2011). Evidence from Cameroon shows that environmental and ecological differences between habitats may be important in the evolution of chimpanzee subspecies. Current evidence about niche differences among the subspecies comes only from habitat suitability models from remote sensing GIS data and is therefore only a starting point for examining the environmental and ecological variation that may contribute to the evolution of chimpanzee populations. In order to place habitat suitability models into a spatial and temporal scale that is ecologically relevant to chimpanzees, it is necessary to ground‐truth them with data regarding local environmental factors, as well as the distribution and availability of resources that chimpanzees rely on for survival.

## 2 MATERIALS AND METHODS

**Figure 2:**
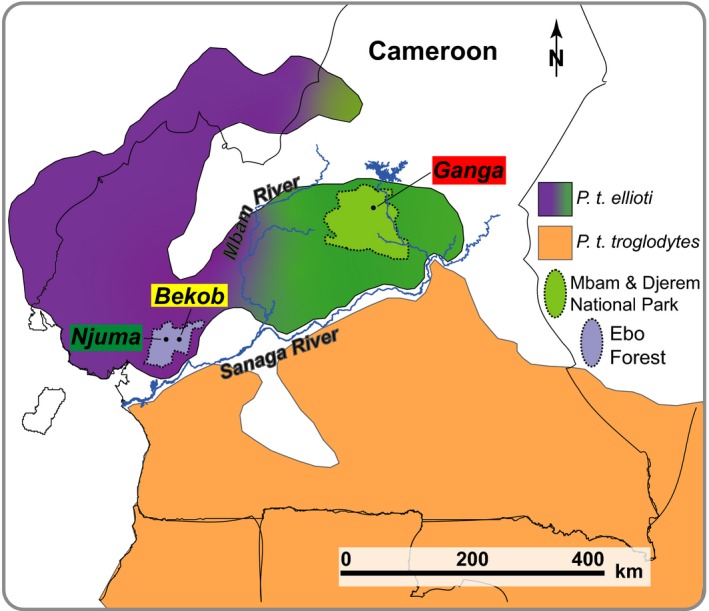
Study sites. The gradient describes the distribution of the two *P. t. ellioti* gene pools: *P. t. ellioti*—rainforest (white) and *P. t. ellioti*—ecotone (gray) west and east of the Mbam River, respectively, in relation to the study sites

We collected fine scale environmental and ecological data including variables reported previously to be important in determining regional differences in chimpanzee socioecology (e.g., Stumpf, 2011) at three locations in Cameroon: Njuma, Bekob, and Ganga (Figure 2). These three sites represent each of the two gene pools in *P. t. ellioti*: in a mostly mature rainforest at Ebo (Njuma), at a rainforest location at Ebo that was heavily modified by small stakeholder agriculture until it was abandoned in the 1960s (Bekob), and finally, in a forest–woodland–savanna ecotone at Mbam & Djerem National Park (MDNP: Ganga).

### 2.1 Study sites

#### 2.1.1 Ebo forest

The Ebo Forest is in the Littoral Region, Cameroon, and extends for more than 1,500 km^2^, of which approximately 1,200 km^2^ is proposed as a national park. With a conservative estimate of at least 500 individuals of the 3,500–9,000 remaining wild individuals, the Ebo Forest harbors an exceptionally important population of *P. t. ellioti* (Morgan et al., 2011) associated with the *P. t. ellioti* (Rainforest) gene pool (Mitchell, Locatelli, Ghobrial, et al., 2015). The forest is characterized by closed‐ and open‐canopy semideciduous and evergreen lowland and submontane rainforest of the Atlantic forest dominated by Fabaceae (Letouzey, 1985). The Ebo Forest also harbors a rich assemblage of other diurnal primates including drills (*Mandrillus leucophaeus*), Preuss's red colobus (*Piliocolobus preussi*), Preuss's monkeys (*Allochrocebus preussi*), and gorillas (*Gorilla gorilla* spp.) (Morgan et al., 2011; Morgan, Wild, & Ekobo, 2003; Oates, Bergl, & Linder, 2004). The forest is also noted for its botanical diversity with several plant endemics including *Palisota ebo*, *Ardisia ebo*, *Inversodicraea ebo*, *Talbotiella ebo,* and *Gilbertiodendron ebo* (van der Burgt, Mackinder, Wieringa, & Estrella, 2015; Cheek et al., 2017; Cheek & Xanthos, 2012; Mackinder, Wieringa, & Burgt, 2010). The main threats to wildlife in Ebo Forest include poaching and the bushmeat trade as well as habitat loss from logging, subsistence‐shifting agriculture and agro‐industrial plantations (Morgan et al., 2011).

We selected two sites in Ebo Forest‐based differences in anthropogenic modification (high: Bekob, and low: Njuma) but having relatively high densities of *P. t. ellioti*: 0.67 chimpanzees km^−2^ (0.44–1.04, 95% CI) following a standing crop nest count method (Ndimbe, Morgan, Marino, & Abwe, 2016) (Figure 2). Njuma is to the west of the Ebo River that traverses the forest from north to south and is composed of closed‐canopy lowland and submontane rainforest that was selectively logged in the late 1980s (Abwe, 2018). Bekob is located approximately 20 km east of Njuma and harbored villages that were relocated in the late 1950s following civil strife at Cameroon's independence (Dowsett‐Lemaire & Dowsett, 2001). Open‐canopy forests at Bekob characterize abandoned villages and farmland in lower altitudes (~500 m above sea level) while higher altitudes (up to 1,200 m) harbor closed‐canopy submontane vegetation (Abwe, 2018; Dowsett‐Lemaire & Dowsett, 2001).

#### 2.1.2 Mbam & Djerem National Park

Mbam & Djerem National Park (MDNP) is located >200 km northeast of Ebo and straddles the Adamawa, Centre and East Regions of Cameroon; and extends over 4,165 km^2^ (Figure 2). The park was created in 2000 as an offset to the environmental impact of the Chad‐Cameroon pipeline project (Moynihan et al., 2004). The MDNP harbors more than 500 individuals of *P. t. ellioti* (Kamgang et al., 2018; Morgan et al., 2011), with a density of 0.33 chimpanzees km^−2^ (0.12–0.86 CI) (Kamgang et al., 2018) and is associated with *P. t. ellioti* (Ecotone) gene pool (Mitchell, Locatelli, Ghobrial, et al., 2015). The park harbors 12 other primate species including gray‐cheeked mangabeys (*Lophocebus albigena*), olive baboons (*Papio anubis*), guereza colobus (*Colobus guereza*), putty‐nosed monkeys (*Cercopithecus nictitans*), and crowned guenons (*C. pogonias*) (Maisels, Ambahe, Ambassa, & Fotso, 2007; Maisels, Fotso, & Hoyle, 2000). The vegetation of the MDNP is a mosaic of forest–woodland–savanna (Maisels et al., 2000). The main conservation threats at MDNP include illegal bushfires, cattle grazing, poaching, and fishing (Maisels et al., 2000). Data were collected at Ganga in the northeast of the park, situated along the Djerem River (Figure 2).

### 2.2 Data collection

Based on the previous studies of habitat suitability and niche differentiation among chimpanzee populations in Cameroon (Sesink Clee et al., 2015), as well as studies of chimpanzee socioecology from other areas of Africa (e.g., Stumpf, 2011), we predicted that (a) the ecotone would have less rainfall volume and seasonality compared to the rainforest, (b) there would be greater variation in plant species diversity within habitats at the ecotone site, (c) plant species diversity would be higher in the rainforest than ecotone, (d) the availability of fleshy fruits would be higher in the rainforest than the ecotone, (e) there would be greater seasonality in fleshy fruit availability at the ecotone than the rainforest, and (f) the incidence of introduced and secondary forest species would be higher at Bekob due to anthropogenic modification. Thus, we designed our data collection to allow us to examine these variables at a fine scale at each of the three study sites.

#### 2.2.1 Climate

Rainfall data were collected daily at ~7.00H from January 2010 to December 2016 at Bekob and Njuma in Ebo using traditional rain gauges by Ebo Forest Research Project. At MDNP, rainfall data over the same period were obtained from the Cameroon Electricity Corporation service at Mbakaou, at the northern border of the park. We categorized the dry season as successive months with <100 mm cumulative rainfall each, and the wet season as successive months with >100 mm cumulative rainfall each (Willie, Tagg, Petre, Pereboom, & Lens, 2014).

#### 2.2.2 Botanical inventory

To assess plant species diversity, we established 10 transects of 2 km length each perpendicular to the main drainage and transects followed a fixed bearing per site: Bekob (270°), Njuma (20°), and Ganga (270°). We enumerated, measured the diameter at breast height (DBH ~1.3 m), and identified all trees and lianas (Bekob: 5,482, Njuma: 5,017, Ganga: 4,908) with a DBH ≥10 cm on a 5 m band (2.5 m on either side of the transect center‐line). Where it was not possible to measure DBH, for example, tall buttressed trees, the diameter was estimated to the nearest 5 cm. From the DBH, we calculated the basal area for trees assuming circular cross‐section of trunks (Morgan, 2001). We used The Plant List (2013) database (http://www.theplantlist.org/) for taxonomic classification.

#### 2.2.3 Potential chimpanzee food resources

We assessed the basal area (BA) of woody plants at each site from trees and lianas ≥10 cm DBH along transects. We further determined the BA of tree species that were potentially important in chimpanzee diets (based on macroscopic fecal analysis). Finally, we determined the stem density of tree species whose fruits were recurrent in chimpanzee diets (based on macroscopic fecal analysis) from trees/lianas ≥10 cm DBH along transects at each site (Potts et al., 2009; Worman & Chapman, 2006). Given the seasonal differences in the fruiting phenology of different species, we compared the frequency of plant species with synchronous and asynchronous fruiting patterns during the wet and/or dry seasons (Potts et al., 2009) across the sites.

#### 2.2.4 Terrestrial herbaceous vegetation (THV)

We assessed THV species in 2 × 2 m quadrats positioned on alternate sides of each transect at 100 m intervals (Morgan, 2001). Given that chimpanzees feed preferentially on THV species from the Marantaceae and Zingiberaceae families (Tutin et al., 1991), we noted the presence/absence of species from these families in each quadrat.

#### 2.2.5 Fruit availability

We assessed fruit availability monthly by counting fallen fruits (including partly eaten and rotting fruits) within a 1 m band along transects (Furuichi, Hashimoto, & Tashiro, 2001) across the three sites between January 2016 and March 2017. All fallen fruits within this band were identified to species or genus level and photographed. Fruit species that were recurrent in chimpanzee diets based on macroscopic fecal analysis were quantified in terms of number of fallen fruits per hectare. To account for seasonality in fruit availability, we distinguished between fruitfall for dry (Bekob & Njuma: December to February, Ganga: December to March) and wet (Bekob & Njuma: March to November, Ganga: April to October) seasons.

#### 2.2.6 Data analysis

We assembled rainfall and ecological variables and completed a principal component analysis (PCA) to infer the variables that were most important in distinguishing each of these three sites from one another. The PCA was completed using R3.4.3 (R Core Team, 2017) to infer the environmental and ecological variables that contribute to the differentiation among the habitats available to chimpanzees at Njuma, Bekob, and Ganga. Variables included in the analysis were annual rainfall volume and seasonality, and ecological data from transects including tree stem density, liana stem density, number of tree species, mean tree size (diameter), basal area for all tree species, basal area for tree species that were recurrent in chimpanzee diet at each site, dry and wet season fruit availability, and frequency of THV in the Marantaceae and Zingiberaceae families in quadrats.

We also carried out pairwise comparisons for each variable to further distinguish the sites from one another. We calculated measures of species diversity including Jaccard Classic and Shannon Diversity indices in EstimateS, version 9.1.0 (Colwell, 2016). We used the Jaccard Classic index to assess variation in species composition among transects/habitats (beta diversity) across each site, and Shannon Diversity index for species diversity (alpha diversity) among the sites (Magurran, 2013). We generated species accumulation curves to depict species richness in relation to sampling effort across the three sites (Gotelli & Colwell, 2001). We used nonparametric Kruskal–Wallis one‐way analysis of variance (ANOVAs) to test for overall habitat differences among the sites including rainfall, plant species and habitat diversity, and fruit availability. We adjusted significant values for multiple comparisons by using the Bonferroni correction. Mann–Whitney *U* tests were used to test for intrasite seasonality in fruit availability.

## 3 RESULTS

### 3.1 Main factors distinguishing the three sites

**Figure 3:**
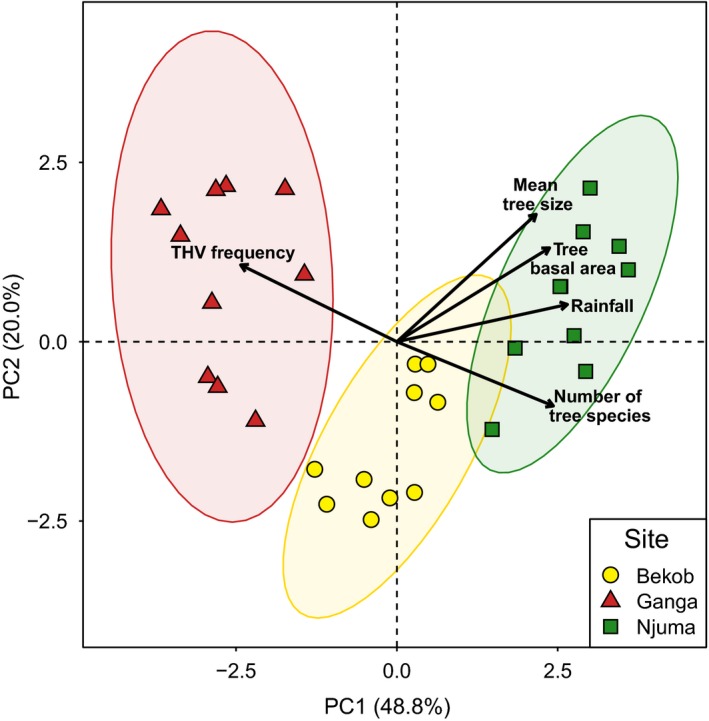
Principal component analysis for ecological characteristics of all sites. Data were collected along ten 2‐km transects at each of the three chimpanzee habitats: Ganga (ecotone), Bekob (human‐modified rainforest), and Njuma (mature rainforest). Biplot arrows show the five most important environmental and ecological components distinguishing chimpanzee habitats

In total, PC1 and PC2 accounted for 68.8% intersite variation (Figure 3). There was a primary separation between the rainforest and the ecotone along PC1 that accounted for 48.8% of the variation. The five components that contributed most to the differentiation of the sites along PC1 included rainfall (accounting for 38.9% of the variation between sites), number of tree species (35.7%), THV frequency (35.6%), basal area of trees (34.8%), and tree size (31.8%). Distinction between the sites in terms of rainfall was characterized by higher annual rainfall at Njuma (mature rainforest) than at Ganga (ecotone). Bekob (human‐modified rainforest) received an intermediate amount of rainfall on average through the study period.

The number of tree species along transects at Njuma and Bekob was higher than at Ganga, while the frequency of THV stems (Marantaceae and Zingiberaceae) was higher in quadrats at Ganga and distinguished the ecotone from the rainforest sites. The separation among the sites along PC2 was linked mainly to tree stem density (42.0%) and liana density (39.0%). The density of tree stems in transects across Bekob and Njuma (rainforest) was higher than at Ganga (ecotone), while the density of lianas was higher for transects at the ecotone than the rainforest sites. To further ascertain inter‐site differences, we carried out pairwise analysis of environmental and ecological variables among and within the sites (Table 1).

**Table 1:** Summary of variation in environmental and ecological variables, and measures of species and habitat diversity (alpha diversity and beta diversity using Shannon and Jaccard indices, respectively) across the rainforest (Bekob and Njuma) and ecotone (Ganga)

Variable	Bekob	Njuma	Ganga	*p*‐value
Rainfall	2,336	3,135	2,173	0.015
Number of tree stems	5,482	5,017	4,908	
Number of families	62	54	42	
Number of species	301	306	184	
Alpha diversity	4.28	4.35	3.73	0.001
Beta diversity	0.37	0.41	0.44	0.001
Basal area of all trees	323.87	511.74	300.47	0.001
Basal area—most consumed fruit species	4.53	4.80	4.40	0.548

### 3.2 Intersite variation in key factors distinguishing the three sites

Overall, the difference in mean monthly rainfall between the rainforest and ecotone was statistically significant: (Kruskal–Wallis: *N* = 252, *X* ^2 ^= 8.410, *df *= 2, *p* = 0.015). Mean monthly rainfall was lower at Ganga than Njuma (Mann–Whitney *U*: *N* = 168, *Z *= −2.767, *p* = 0.017). There was no significant difference between Bekob and Njuma (*p* = 0.098), and between Ganga and Bekob (Table 1). The wet season at Bekob and Njuma extended between February‐March and November, and for Ganga between March‐April and October (Supporting Information Figure S1).

**Figure 4:**
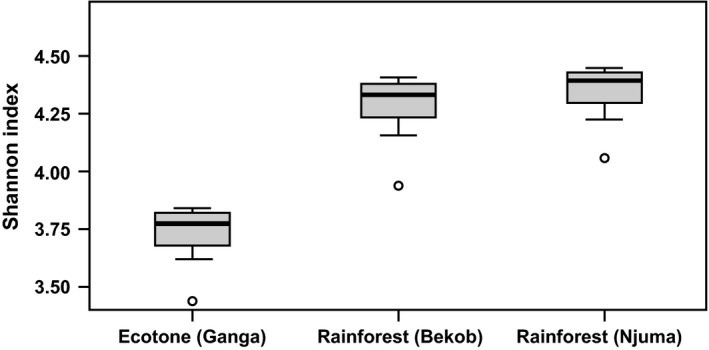
Variation in tree species richness (alpha diversity) among Ganga, Bekob, and Njuma using the Shannon Diversity Index measured from tree species along the 10 transects at each site

**Figure 5:**
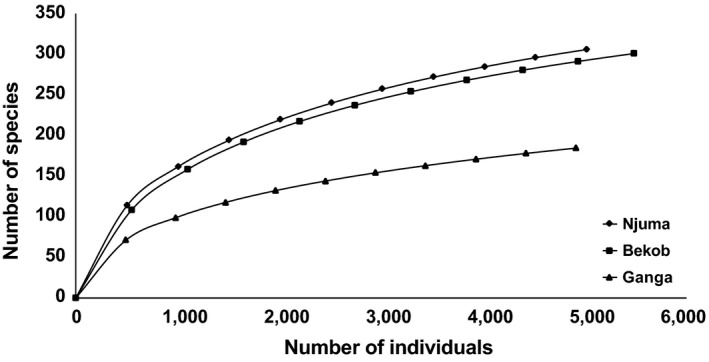
Tree species richness (accumulation curves) in relation to sampling effort across 10 botanical transects per site: Bekob (5,482 trees), Njuma (5,017 trees), and Ganga (4,908 trees). The species accumulation curves did not asymptote, suggesting the need for a larger sample size

**Figure 6:**
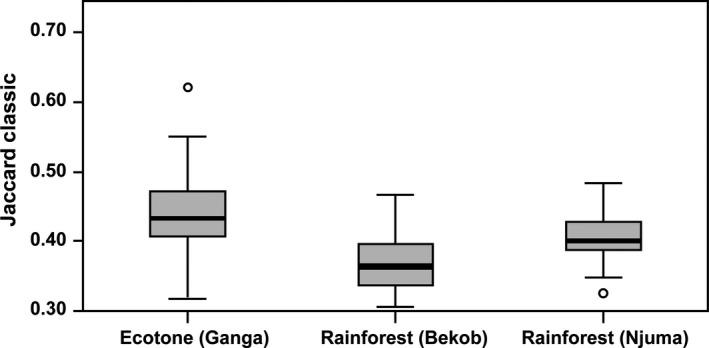
Variation in tree species composition (beta diversity) among transects/habitats in Ganga, Bekob, and Njuma using the Jaccard Classic index

The number of tree families and species was higher at Bekob and Njuma than Ganga (Supporting Information Tables S1–S3). In terms of measures of species diversity among and within the sites, tree species diversity (alpha diversity) was higher for transects in the rainforest than the ecotone (Figures 4 and 5), while variation in plant species composition among habitats/transects within each site (beta diversity) was higher for the ecotone than the rainforest (Figure 6). The basal area for trees across the sites was significantly higher at Njuma (mature rainforest) than at Bekob (human‐modified rainforest) and Ganga (ecotone). However, there was no difference among the sites in the basal area nor the stem density of fruiting tree species that were commonly consumed by chimpanzees at each site.

The frequency of THV of the Marantaceae and Zingiberaceae families in quadrats was higher at the ecotone than the rainforest sites. Marantaceae species occurred in 11 quadrats at Bekob (5.5%), 14 at Njuma (7.1%), and 88 at Ganga (44%), while Zingiberaceae species occurred in 17 quadrats at Bekob (I8.5%), 3 at Njuma (1.5%), and 55 at Ganga (27.5%). The encounter ratio of Marantaceae species stems as compared to other THV and saplings was 1:120 for Bekob, 1:68 for Njuma, and 1:14 for Ganga. The encounter ratio of Zingiberaceae species stems to other THV and saplings was 1:57 for Bekob, 1:340 for Njuma, and 1:26 for Ganga.

**Figure 7:**
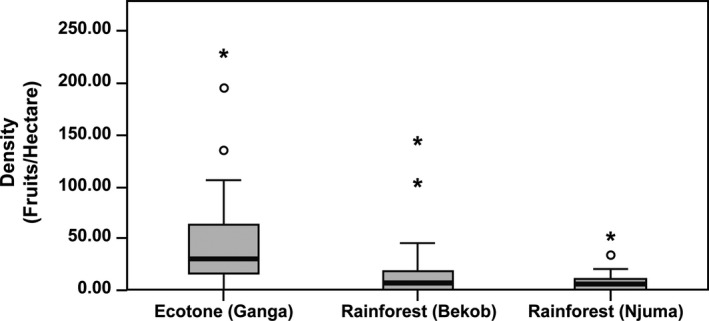
Variation in fruit availability for Bekob and Njuma (rainforest) and Ganga (ecotone) using monthly fruitfall for fruit species that were most represented in chimpanzee diets (based on macroscopic fecal sample analysis) from January 2016 to March 2017

The overall density of fruitfall was higher at Ganga than at Njuma (*Z* = 3.553, *p* < 0.001) and at Bekob (*Z *= −2.653, *p* = 0.024). There was no difference in fruitfall between the two rainforest sites (Figure 7). When we examined intrasite difference in seasonal fruitfall, there was no significant seasonal difference in fruit availability at Bekob (*N* = 23, Mann–Whitney *U* test: *Z* = 89.00, *p* = 0.169). Fruit availability was higher during the wet compared to the dry season at Njuma (*N* = 26, *Z* = 126.500, *p* = 0.012) and Ganga (*N* = 19, *Z* = 72.000, *p* = 0.010; Supporting Information Table S4).

## 4 DISCUSSION

In this study, we examined the specific abiotic factors and biotic conditions predicted by niche tests from ENM comparisons that differentiate two distinct gene pools of *P. t. ellioti* in Cameroon in two rainforest locations and one ecotone habitat at a fine geographic scale using chimpanzee‐relevant variables. We compared environmental and ecological variables across Ebo Forest (rainforest) and MDNP (ecotone). We also examined differences between two rainforest sites in Ebo Forest that contrasted in levels of anthropogenic modification (high: Bekob, and low: Njuma) to understand how agriculture might also affect chimpanzee natural resource density and their potential utilization of human‐modified landscapes. The impact of both factors on suitable chimpanzee habitat might be lost by considering ecotones and agricultural lands solely as unsuitable habitats, which is often an underlying assumption of ape ENMs (Junker et al., 2012).

We first examined rainfall volume among sites, which was a key variable that distinguished the rainforest and ecotone habitats from one another in ENMs (Sesink Clee et al., 2015) and was also linked with annual variation in resources available to chimpanzees at local and species‐wide scales (Stumpf, 2011). Our detailed comparisons among Bekob, Njuma, and Ganga were consistent with previously published comparisons of chimpanzee ENMs (Sesink Clee et al., 2015). The PCA revealed that annual rainfall was one of the main distinguishing variables between the rainforest and ecotone habitats. Rainfall was lower at Ganga (ecotone) compared to Njuma and to a lesser extent Bekob (both rainforest). Rainfall patterns across the year were similar, with all sites having one dry and one wet season annually. The length of the rainy season varied between the rainforest and ecotone sites. Bekob and Njuma had about three months of dry season compared to four‐five months at Ganga. The *P. t. ellioti* habitat at Gashaka Gumti National Park (GGNP), Nigeria, has a similar rainfall pattern as Ganga (Hohmann, Fowler, Sommer, & Ortmann, 2012; Sommer, Adanu, Faucher, & Fowler, 2004). The wide range of chimpanzee habitats across Africa is characterized by variation in rainfall volume and seasonal patterns (Stumpf, 2011). Equatorial rainforest habitats receive more rainfall, associated with more marked seasonality (Chapman, Chapman, Wrangham, Isabirye‐Basuta, & Ben‐David, 1997; Chapman, Wrangham, & Chapman, 1994; Hemingway & Bynum, 2005; van Schaik & Brockman, 2005; Stumpf, 2011). Rainfall amounts are lower at more tropical woodland and savanna habitats and associated with lower seasonality (Hunt & McGrew, 2002; McGrew, Marchant, & Nishida, 1996; Pruetz & Bertolani, 2009; Tutin et al., 1991). Differences in rainfall volume and seasonality across habitats are linked with variation in plant species diversity and fruiting patterns, which influence many aspects of local chimpanzee socioecology (Doran, Jungers, Sugiyama, Fleagle, & Heesy, 2002; Hunt & McGrew, 2002; Knott, 2005; McGrew et al., 1988; Murray, Eberly, & Pusey, 2006; Pruetz & Bertolani, 2009; Wrangham, Chapman, Clark‐Arcadi, & Isabirye‐Basuta, 1996).

Habitat diversity is a function of environmental conditions including rainfall (Chapman, Olson, & Trumm, 2004; Hohmann et al., 2012), relief (Nkurunungi, Ganas, Robbins, & Stanford, 2004; Proctor, Edwards, Payton, & Nagy, 2007), soil moisture (Marshall et al., 2009), and anthropogenic influence (Arnhem, Dupain, Vercauteren Drubbel, Devos, & Vercauteren, 2007; Chapman, Balcomb, Gillespie, Skorupa, & Struhsaker, 2000). Habitat heterogeneity can be advantageous to frugivorous primates when the different categories are rich in plant species that can reduce the incidence of seasonality in fruit availability. Thus, we next investigated habitat diversity within each of the sites. As predicted, there was greater variation in tree species diversity among habitats at the ecotone compared to the rainforest. Within the rainforest sites, there was greater tree species diversity among habitats at Njuma (mature rainforest) than Bekob (human‐modified site). Variation in tree species among habitats at Ganga could be linked to environmental conditions and anthropogenic modification including annual bushfires (Mitchard, Saatchi, Gerard, Lewis, & Meir, 2009). At Ganga, closed‐canopy habitats were associated with lowland rainforest species including *Pseudospondias microcarpa*, *Uapaca guineensis*, *Canarium schweinfurthii*, while open‐ and closed‐canopy secondary and colonizing habitats were rich in *Myrianthus arboreus* and various species of *Landolphia*, *Saba* and *Ficus *(Supporting Information Tables S1–S3). Species variation within habitats at Njuma and Bekob could be linked to the wide altitudinal range, spanning lowland and submontane vegetation classes. Relics of the recent anthropogenic history at the Bekob included the prevalence of introduced and secondary forest species at lower altitudes. At Bekob, secondary forest habitats harbored several species including *Musanga cecropioides* (umbrella tree), *Elaeis guineensis* (oil palm), and *Dacryodes *spp. that are important in chimpanzee diets.

In general, tree species diversity was higher at Bekob and Njuma than at Ganga, consistent with the prediction of higher plant species diversity in rainforest than ecotone habitats. Climatic conditions including the length of the wet season were less variable at the rainforest than at the ecotone, and the wider altitudinal range at Bekob and Njuma supported lowland and submontane plant species, respectively. Environmental conditions at MDNP are similar to GGNP, Nigeria, and additionally, tree species richness was similar at both sites (Fowler, 2006). Sites with high species diversity are generally linked with greater fleshy fruit diversity in chimpanzee diets (Head et al., 2011; Newton‐Fisher, 1999; Potts, Watts, & Wrangham, 2011; Tutin et al., 1991; Tweheyo & Lye, 2005; Watts et al., 2012) than sites with lower species diversity (Chancellor, Rundus, & Nyandwi, 2012; Hunt & McGrew, 2002; Stanford & Nkurunungi, 2003). The density of lianas was more important at the ecotone compared to the rainforest sites. Fruits from many lianas including *Landolphia* spp. and *Saba* spp. are important food sources for chimpanzees (Moscovice et al., 2007; Piel et al., 2017). The frequency and diversity of Marantaceae and Zingiberaceae species were higher at the ecotone and distinguished the site from the rainforest. These are examples of terrestrial herbs, which are also important in chimpanzee diets especially during periods of fruit scarcity (Boesch, Hohmann, & Marchant, 2002; Tutin et al., 1997; Yamakoshi, 2004).

Other structural differences between rainforest and ecotone, and within rainforest habitats were related to tree size and stem density. Tree size and basal area were larger at Njuma than either Bekob or Ganga. However, tree stem density was higher at Bekob than at Njuma and Ganga. These differences could be attributed to the degree of human modification. Most of the lower altitude vegetation at Bekob was relatively young with smaller tree sizes at various stages of ecological succession due to recent anthropogenic modification. Lower stem density and basal area at Ganga could be attributed to climatic conditions and anthropogenic influence including annual bushfires.

Fruit availability was another key component that distinguished the sites from one another. Counter to our prediction, there were more fleshy fruits from trees and lianas available in the ecotone than the rainforest. Higher fruit availability at the ecotone could be linked to swamps along the main rivers and irradiance. The flood zone of the Djerem River and its tributaries seasonally irrigate swamps that store moisture that could alter the effects of the longer dry seasons at Ganga (Maisels et al., 2000). These swamps could also be very fertile due to alluvial deposits from annual floods, but this was not tested. In addition, more open habitats characteristic of the ecotone may benefit from higher irradiance, providing for greater fruit ripening in upper‐ and lower‐canopy species. Fruit production by trees and lianas at GGNP, an ecotone habitat, was higher than at Salonga, a rainforest habitat in DR Congo (Hohmann et al., 2012).

However, there was more marked seasonality in fruit availability at Ganga, consistent with the prediction of greater seasonality in fruit availability at the ecotone compared to the rainforest. The wet season at Ganga was associated with higher fruit availability with many tree and liana species fruiting synchronously. Conversely, fruit phenology in the dry season at Ganga was low and limited to a few species with asynchronous fruiting patterns. There was less marked seasonality in fruit availability at Bekob and Njuma, where many species produced fruits synchronously during the dry and wet seasons, including several species that fruited asynchronously. At Bekob, *E. guineensis*, *M. cecropioides* and other human‐introduced and secondary forest plant species produced fruits asynchronously in the wet and dry seasons.

Chimpanzees are fruit specialists and their socioecology is largely influenced by the spatial and temporal distribution of fleshy fruits (Anderson, Nordheim, Boesch, & Moermond, 2002; Mitani, Watts, & Lwanga, 2002). Given the greater seasonality in fleshy fruit availability, the chimpanzee communities at the ecotone may be subjected to greater seasonal shifts in dietary components compared to the rainforest. The consumption of fallback food resources including THV, vertebrates, and invertebrates may be more frequent and consistent at the ecotone than the rainforest sites, especially during the dry season. The consumption of vertebrates and invertebrates by *P. t. ellioti* at Ngel Nyaki, Nigeria, increased during the dry season. The dry season was marked by lower variety in fleshy fruit availability (Dutton & Chapman, 2015), suggesting that this could be a fallback food strategy for chimpanzees at Ngel Nyaki. Seasonality in fruit availability is a major determinant of chimpanzee grouping patterns as it is linked with other determinants including female cycling (Anderson et al., 2002; Mitani et al., 2002; Wallis, 1995). Low availability and/or patchy distribution of fruits increase ranging and grouping costs (Chapman, Wrangham, & Chapman, 1995; Wrangham et al., 1996), and chimpanzees at the ecotone may be subjected to wider ranging and less group cohesion during the dry season. Low fruit availability was associated with lower rates of gregariousness in *P. t. ellioti* at GGNP (Hohmann et al., 2012). Thus, foraging parties are expected to be smaller at the ecotone during the dry season. Party sizes may be larger and more stable at the rainforest sites due to lower seasonal variation in fleshy fruit availability. Introduced and secondary forest species including *E. guineensis* and *M. cecropioides* could play an important role in the diets of chimpanzees at Bekob, the human‐modified site. Inter‐ and intrasite differences in tree species richness and seasonality in fleshy fruit availability may also influence nesting behavior including nest site selection, nest group size, and nesting tree choice. The location of nesting sites at Ganga (ecotone) may reflect seasonal variation in fruit availability compared to the rainforest with less seasonality, while nesting tree preferences would be site specific.

These results affirm the diversity and distinctiveness in modeled *P. t. ellioti* habitats in Cameroon (Sesink Clee et al., 2015). Rainforests are often presumed to be the “ideal” chimpanzee habitat, with most ENM studies positing that intact forests are necessary for chimpanzee survival, and that ecotones and anthropogenically modified sites are not suitable for sustaining large, healthy populations (Sesink Clee et al., 2015; Strindberg et al., 2018). Our results however show that these “marginal” habitats have the resources to sustain large chimpanzee populations, a fact that would be lost with large scale habitat suitability models. Harboring more than 500 individuals each of the most threatened chimpanzee subspecies, the Ebo Forest (rainforest) and the Mbam & Djerem National Park (ecotone) are important strongholds for *P. t. ellioti* (Kamgang et al., 2018; Morgan et al., 2011). Despite their proximity, there are structural differences between Bekob and Njuma linked to anthropogenic modification, but chimpanzee densities are high at both sites. Behavioral diversity among and within these populations is linked to habitat variation (Abwe, 2018). Fleshy fruits are the most important dietary component in chimpanzee populations across these sites, but the diversity and seasonality of fruit consumption vary. The rainforest chimpanzee populations consistently consume more fleshy fruit species throughout the year, but the incidence of nonfruit plant parts in their diet is higher in the wet season. The consumption of fleshy fruits is higher for the ecotone chimpanzees during the wet season, while the dry season is associated with a higher reliance on nonfruit plant parts including THV. The incidence of introduced and secondary forest fruit species including *E. guineensis* and *M. cecropioides* in chimpanzee diets is higher for the population at Bekob (human‐modified rainforest), especially during periods of fleshy fruit scarcity. The consumption of meat including mammals, ants, and termites is higher at the ecotone and is more marked during the dry season (Abwe, 2018). Closed‐canopy vegetation and steep relief were linked to nesting site location for the rainforest chimpanzees, while fruit availability was related to chimpanzee nesting site selection at the ecotone. Nest group sizes for the ecotone were smaller than for the rainforest chimpanzees. However, larger nest groups were associated with the wet season (higher fruit availability) at the ecotone.

We speculate that adaptations to local ecological conditions including seasonality in fruit availability may be important in promoting genetic diversity within the subspecies in rainforest and ecotone habitats, as it has already been shown that sex‐specific patterns of community and population structure are markedly different across the rainforest and ecotone (Mitchell et al., 2018). These important chimpanzee populations and their habitats are increasingly threatened by hunting and the bushmeat trade, habitat destruction linked to subsistence and agro‐industrial plantations, pet trade, as well as climate change (Morgan et al., 2011; Sesink Clee et al., 2015). These observations suggest the need for a more realistic landscape planning approach to conservation planning for the remnant populations of the species.

## CONFLICT OF INTEREST

The authors declare they have no conflict of interest.

## AUTHORS’ CONTRIBUTIONS

EEA, BF, AM, RCF, BJM, and MKG designed the study and developed the methodology. EEA, FB, BT, RD, MEK, ET, and RA collected the data. EEA, DMV, MWM, and MKG performed the analysis. EEA and MKG led the writing of the manuscript. All authors contributed critically to the drafts and gave final approval for publication.

## Data Availability

Data will be deposited at the Global Biodiversity Information Facility.

## References

[ece34871-bib-0001] Abwe, E. E. (2018). Linking behavioral diversity with genetic and ecological variation in the Nigeria‐Cameroon chimpanzee (Pan troglodytes ellioti). Philadelphia, PA: Drexel University.

[ece34871-bib-0002] Anderson, D. P. , Nordheim, E. V. , Boesch, C. , & Moermond, T. (2002). Factors influencing fission‐fusion grouping in chimpanzees in the Taï National Park, Côte d’Ivoire. Behavioural diversity in chimpanzees and bonobos (pp. 90–101). Cambridge: Cambridge University Press.

[ece34871-bib-0003] Anderson, D. P. , Nordheim, E. , Moermond, T. C. , Bi, G. , Zoro, B. , & Boesch, C. (2005). Factors influencing tree phenology in Taï National Park, Côte d'Ivoire. Biotropica, 37, 631–640. 10.1111/j.1744-7429.2005.00080.x.

[ece34871-bib-0004] Arnhem, E. , Dupain, J. , Vercauteren Drubbel, R. , Devos, C. , & Vercauteren, M. (2007). Selective logging, habitat quality and home range use by sympatric gorillas and chimpanzees: A case study from an active logging concession in southeast Cameroon. Folia Primatologica, 79, 1–14. 10.1159/000107664. 17726332

[ece34871-bib-0005] Basabose, A. K. , & Yamagiwa, J. (2002). Factors affecting nesting site choice in chimpanzees at Tshibati, Kahuzi‐Biega National Park: Influence of sympatric gorillas. International Journal of Primatology, 23, 263–282. 10.1023/A:1013879427335.

[ece34871-bib-0006] Boesch, W. , (1996). Social grouping in Tai chimpanzees In W., McGrew , L. F., Marchant , & C., Boesch (Eds.), Great ape societies (pp. 101–113). Cambridge: Cambridge University Press.

[ece34871-bib-0007] Boesch C., Hohmann G., & Marchant L. F. (Eds.) (2002). Behavioural diversity in chimpanzees and bonobos. Cambridge: Cambridge University Press.

[ece34871-bib-0008] Brownlow, A. R. , Plumptre, A. J. , Reynolds, V. , & Ward, R. (2001). Sources of variation in the nesting behavior of chimpanzees (Pan troglodytes schweinfurthii) in the Budongo forest, Uganda. American Journal of Primatology, 55, 49–55. 10.1002/ajp.1038[pii]10.1002/ajp.1038. 11536316

[ece34871-bib-0009] Caldecott, J. O. , & Miles, L. (2005). World atlas of great apes and their conservation. Los Angeles, London: University of California Press Berkeley.

[ece34871-bib-0010] Chancellor, R. , Rundus, A. , & Nyandwi, S. (2012). The influence of seasonal variation on chimpanzee (Pan troglodytes schweinfurthii) fallback food consumption, nest group size, and habitat use in Gishwati, a montane rain forest fragment in Rwanda. International Journal of Primatology, 33, 115–133. 10.1007/s10764-011-9561-4.

[ece34871-bib-0011] Chapman, C. A. , Balcomb, S. R. , Gillespie, T. R. , Skorupa, J. P. , & Struhsaker, T. T. (2000). Long-term effects of logging on African primate communities: A 28-year comparison from Kibale National Park, Uganda. Conservation Biology, 14, 207–217. 10.1046/j.1523-1739.2000.98592.x

[ece34871-bib-0012] Chapman, C. A. , Chapman, L. J. , Wrangham, R. , Isabirye‐Basuta, G. , & Ben‐David, K. (1997). Spatial and temporal variability in the structure of a tropical forest. African Journal of Ecology, 35, 287–302. 10.1111/j.1365-2028.1997.083-89083.x.

[ece34871-bib-0013] Chapman, C. A. , Chapman, L. J. , Zanne, A. E. , Poulsen, J. R. , & Clark, C. J. (2005). A 12-Year phenological record of fruiting: Implications for frugivore populations and indicators of climate change In Dew J. L., & Boubli J. P. (Eds.), Tropical fruits and frugivores (pp. 75–92). Dordrecht: Springer.

[ece34871-bib-0014] Chapman, C. A. , Wrangham, R. , & Chapman, L. J. (1994). Indices of habitat‐wide fruit abundance in tropical forests. Biotropica, 26, 160–171. 10.2307/2388805.

[ece34871-bib-0015] Chapman, C. A. , Wrangham, R. W. , & Chapman, L. J. (1995). Ecological constraints on group size: An analysis of spider monkey and chimpanzee subgroups. Behavioral Ecology and Sociobiology, 36, 59–70. 10.1007/BF00175729.

[ece34871-bib-0016] Chapman, H. M. , Olson, S. M. , & Trumm, D. (2004). An assessment of changes in the montane forests of Taraba State, Nigeria, over the past 30 years. Oryx, 38, 282–290. 10.1017/S0030605304000511.

[ece34871-bib-0017] Cheek, M. , Feika, A. , Lebbie, A. , Goyder, D. , Tchiengue, B. , Sene, O. , … Van DerBurgt, X. (2017). A synoptic revision of Inversodicraea (Podostemaceae). Blumea‐Biodiversity, Evolution and Biogeography of Plants, 62, 125–156. 10.3767/blumea.2017.62.02.07.

[ece34871-bib-0018] Cheek, M. , & Xanthos, M. (2012). Ardisia ebo sp. nov. (Myrsinaceae), a creeping forest subshrub of Cameroon and Gabon. Kew Bulletin, 67, 281–284. 10.1007/s12225-012-9362-8.

[ece34871-bib-0019] Colwell, R. (2016). EstimateS: Statistical estimation of species richness and shared species from samples, version 9 [M/OL~.

[ece34871-bib-0020] Deblauwe, I. (2009). Temporal variation in insect‐eating by chimpanzees and gorillas in southeast Cameroon: Extension of niche differentiation. International Journal of Primatology, 30, 229 10.1007/s10764-009-9337-2.

[ece34871-bib-0021] Dimiceli, C., Carroll, M. , Sohlberg, R. , Huang, C. , Hansen, M. , & Townshend, J. (2011). Annual global automated MODIS vegetation continuous fields (MOD44B) at 250 m spatial resolution for data years beginning day 65, 2000–2010, collection 5 percent tree cover. College Park, MD: University of Maryland.

[ece34871-bib-0022] Doran, D. M. , Jungers, W. L. , Sugiyama, Y. , Fleagle, J. G. , & Heesy, C. (2002). Multivariate and phylogenetic approaches to understanding chimpanzee and bonobo behavioural diversity In Boesch C., Hohmann G., & Marchant L. F. (Eds.), Behavioural diversity in chimpanzees and bonobos (pp. 14–34). Cambridge; New York: Cambridge University Press.

[ece34871-bib-0023] Dowsett‐Lemaire, F. , & Dowsett, R. (2001). First survey of the birds and mammals of the Yabassi area. South‐western Cameroon: Unpublished report for WWF Cameroon.

[ece34871-bib-0024] Dutton, P. , & Chapman, H. (2015). Dietary preferences of a submontane population of the rare Nigerian‐Cameroon chimpanzee (Pan troglodytes ellioti) in Ngel Nyaki Forest Reserve, Nigeria. American Journal of Primatology, 77, 86–97. 10.1002/ajp.22313. 25231641

[ece34871-bib-0025] Farr, T. G. , Rosen, P. A. , Caro, E. , Crippen, R. , Duren, R. , Hensley, S. , … Roth, L. (2007). The shuttle radar topography mission. Reviews of Geophysics, 45(2). 10.1029/2005rg000183.

[ece34871-bib-0026] Fowler, A. (2006). Behavioural ecology of chimpanzees (Pan troglodytes vellerosus) at Gashaka, Nigeria. London: University College London (University of London).

[ece34871-bib-0027] Furuichi, T. , Hashimoto, C. , & Tashiro, Y. (2001). Fruit availability and habitat use by chimpanzees in the Kalinzu Forest, Uganda: Examination of fallback foods. International Journal of Primatology, 22, 929–945. 10.1023/A:1012009520350.

[ece34871-bib-0028] Gonder, M. K. , Disotell, T. R. , & Oates, J. F. (2006). New genetic evidence on the evolution of chimpanzee populations, and implications for taxonomy. International Journal of Primatology, 27, 1103–1127. 10.1007/s10764-006-9063-y.

[ece34871-bib-0029] Gonder, M. K. , Locatelli, S. , Ghobrial, L. , Mitchell, M. W. , Kujawski, J. T. , Lankester, F. J. , … Tishkoff, S. A. (2011). Evidence from Cameroon reveals differences in the genetic structure and histories of chimpanzee populations. Proceedings of the National Academy of Sciences, 108, 4766–4771. 10.1073/pnas.1015422108. PMC3064329 21368170

[ece34871-bib-0030] Gonder, M. K. , Locatelli, S. , Ghobrial, L. , & Sheppard, A. D. (2009). The genetic history of chimpanzees across the Gulf of Guinea region. American Journal of Physical Anthropology, 48, 136.

[ece34871-bib-0031] Gonder, M. K. , Oates, J. F. , Disotell, T. R. , Forstner, M. R. , Morales, J. C. , & Melnick, D. J. (1997). A new west African chimpanzee subspecies? Nature, 388, 337 10.1038/41005 9237749

[ece34871-bib-0032] Gotelli, N. J. , & Colwell, R. K. (2001). Quantifying biodiversity: Procedures and pitfalls in the measurement and comparison of species richness. EcologyLetters, 4, 379–391. 10.1046/j.1461-0248.2001.00230.x.

[ece34871-bib-0033] Head, J. S. , Boesch, C. , Makaga, L. , & Robbins, M. M. (2011). Sympatric chimpanzees (Pan troglodytes troglodytes) and gorillas (Gorilla gorilla gorilla) in Loango National Park, Gabon: Dietary composition, seasonality, and intersite comparisons. International Journal of Primatology, 32, 755–775. 10.1007/s10764-011-9499-6.

[ece34871-bib-0034] Hemingway, C. A. , & Bynum, N. (2005). The influence of seasonality on primate diet and ranging In Brockman D. K., & Schaik C. V. (Eds.), Seasonality in primates: Studies of living and extinct human and non‐human primates (pp. 57–104). Cambridge; New York: Cambridge University Press.

[ece34871-bib-0035] Herbinger, I. , Boesch, C. , & Rothe, H. (2001). Territory characteristics among three neighboring chimpanzee communities in the Tai National Park, Cote d'Ivoire. International Journal of Primatology, 22, 143–167. 10.1023/A:1005663212997.

[ece34871-bib-0036] Hohmann, G. , Fowler, A. , Sommer, V. , & Ortmann, S. (2012). Frugivory and gregariousness of Salonga bonobos and Gashaka chimpanzees: the influence of abundance and nutritional quality of fruit. Feeding ecology in apes and other primates (pp. 123–159). Cambridge: Cambridge University Press NY.

[ece34871-bib-0037] Humle, T. , & Matsuzawa, T. (2001). Behavioural diversity among the wild chimpanzee populations of Bossou and neighbouring areas, Guinea and Cote d’Ivoire, West Africa. Folia Primatologica, 72, 57–68. 10.1159/000049924. 11490130

[ece34871-bib-0038] Hunt, K. D. , & McGrew, W. C. (2002). Chimpanzees in the dry habitats of Assirik, Senegal and Semliki wildlife reserve, Uganda In Boesch C., Hohmann G., & Marchant L. F. (Eds.), Behavioural diversity in chimpanzees and bonobos (pp. 35–51). Cambridge, UK: Cambridge University Press.

[ece34871-bib-0039] Junker, J. , Blake, S. , Boesch, C. , Campbell, G. , Toit, L. , Duvall, C. , … Hjalmar, K. (2012). Recent decline in suitable environmental conditions for African great apes. Diversity and Distributions, 18(11), 1077–1091. 10.1111/ddi.12005.

[ece34871-bib-0040] Kamgang, S. A. , Bobo, K. S. , Maisels, F. , Ambahe, R. D. , Ongono, D. E. , Gonder, M. K. , … Sinsin, B. (2018). The relationship between the abundance of the Nigeria‐Cameroon chimpanzee (Pan troglodytes ellioti) and its habitat: A conservation concern in the Mbam‐Djerem National Park. Cameroon. BMC Ecology, 18, 40 10.1186/s12898-018-0199-3. 30285707 PMC6167774

[ece34871-bib-0041] Knott, C. D. (2005). Energetic responses to food availability in the great apes; implications for hominin evolution In Brockman D. K., & vanSchaik C. P. (Eds.), Seasonality in primates: Studies of living and extinct human and non‐human primates (pp. 351–378). Cambridge: Cambridge University Press.

[ece34871-bib-0042] Letouzey, R. (1985). Notice de la carte phytogeographique du Cameroun au 1: 500,000 (1985).

[ece34871-bib-0043] Mackinder, B. A. , Wieringa, J. J. , & Van Der Burgt, X. M. (2010). A revision of the genus Talbotiella Baker f. (Caesalpinioideae: Leguminosae). Kew Bulletin, 65, 401–420. 10.1007/s12225-010-9217-0.

[ece34871-bib-0044] Magurran, A. E. (2013). Measuring biological diversity. Oxford, UK: Blackwell.

[ece34871-bib-0045] Maisels, F. , Ambahe, R. , Ambassa, E. , & Fotso, R. (2007). New Northwestern range limit of the northern talapoin, Mbam et djerem national park, Cameroon. Primate Conservation, 21, 89–91. 10.1896/0898-6207.21.1.89.

[ece34871-bib-0046] Maisels, F. , Fotso, C. R. , & Hoyle, D. (2000). Mbam Djerem National Park. Conservation status. Large mammals and human impact. New York, NY: Wildlife Conservation Society.

[ece34871-bib-0047] Marshall, A. J. , Ancrenaz, M. , Brearley, F. Q. , Fredriksson, G. M. , Ghaffar, N. , Heydon, M. , Morrogh‐Bernard, H. C. (2009). The effects of forest phenology and floristics on populations of Bornean and Sumatran orangutans In Wich S. A., Atmoko S. U., Setia T. M., & vanSchaik C. (Eds.), P. Orangutans: Geographical Variation in Behavioral Ecology (pp. 97–118). Oxford: Oxford University Press.

[ece34871-bib-0048] McGrew, W. C. , Baldwin, P. J. , & Tutin, C. E. G. (1988). Diet of wild chimpanzees (Pan troglodytes verus) at Mt. Assirik, Senegal: I. Composition. American Journal of Primatology, 16, 213–226. 10.1002/ajp.1350160304. 31968861

[ece34871-bib-0049] McGrew, W. C. , Marchant, L. F. , & Nishida, T. (1996). Great ape societies. Cambridge, New York, NY, USA: Cambridge University Press.

[ece34871-bib-0050] Mitani, J. C. (2006). Demographic influences on the behavior of chimpanzees. Primates, 47, 6–13. 10.1007/s10329-005-0139-7. 16283424

[ece34871-bib-0051] Mitani, J. , Watts, D. , & Lwanga, J. (2002). Ecological and social correlates of chimpanzee party size and composition In Boesch C., Hohmann G., & Marchant L. F. (Eds.), Behavioural diversity in chimpanzees and bonobos (pp. 102–111). Cambridge: Cambridge University Press.

[ece34871-bib-0052] Mitchard, E. T. , Saatchi, S. S. , Gerard, F. , Lewis, S. , & Meir, P. (2009). Measuring woody encroachment along a forest–savanna boundary in Central Africa. Earth Interactions, 13, 1–29. 10.1175/2009EI278.1.

[ece34871-bib-0053] Mitchell, M. W. , & Gonder, M. K. (2013). Primate speciation: A case study of African apes. Nature Education Knowledge, 4(2), 1.

[ece34871-bib-0054] Mitchell, M. W. , Locatelli, S. , Abwe, E. E. , Ghobrial, L. , & Gonder, M. K. (2018). Male‐driven differences in chimpanzee (pan troglodytes) population genetic structure across three habitats in Cameroon and Nigeria. International Journal of Primatology, 39, 581–601. 10.1007/s10764-018-0053-7.

[ece34871-bib-0055] Mitchell, M. W. , Locatelli, S. , Sesink Clee, P. R. , Thomassen, H. A. , & Gonder, M. K. (2015). Environmental variation and rivers govern the structure of chimpanzee genetic diversity in a biodiversity hotspot. BMC Evolutionary Biology, 15, 1 10.1186/s12862-014-0274-0. 25608511 PMC4314796

[ece34871-bib-0056] Mitchell, M. W. , Locatelli, S. , Ghobrial, L. , Pokempner, A. A. , Sesink Clee, P. R. , Abwe, E. E. , … Gonder, M. K. (2015). The population genetics of wild chimpanzees in Cameroon and Nigeria suggests a positive role for selection in the evolution of chimpanzee subspecies. BMC Evolutionary Biology, 15, 3 10.1186/s12862-014-0276-y. 25608610 PMC4314757

[ece34871-bib-0057] Morgan, B. J. (2001). Ecology of mammalian frugivores in the Réserve de Faune du Petit Loango. Gabon: University of Cambridge.

[ece34871-bib-0058] Morgan, B. J. , & Abwe, E. E. (2006). Chimpanzees use stone hammers in Cameroon. Current Biology, 16, R632–R633. 10.1016/j.cub.2006.07.045. 16920608

[ece34871-bib-0059] Morgan, B. J. , Adeleke, A. , Bassey, T. , Bergl, R. , Dunn, A. , Fotso, R. , Williamson, E. (2011). Regional Action Plan for the Conservation of the Nigeria‐Cameroon Chimpanzee (Pan troglodytes ellioti). Gland, Switzerland and San Diego, CA, USA, IUCN/SSC Primate Specialist Group and Zoological Society of San Diego, CA, USA.

[ece34871-bib-0060] Morgan, B. J. , Wild, C. , & Ekobo, A. (2003). Newly Discovered Gorilla Population in the Ebo Forest, Littoral Province, Cameroon. International Journal of Primatology, 24, 1129–1137. doi.:10.1023/A:1026288531361.

[ece34871-bib-0061] Morgan, D. , & Sanz, C. (2006). Chimpanzee feeding ecology and comparisons with sympatric gorillas in the Goualougo Triangle, Republic of Congo In Hohmann G., Robbins M., & Boesch C. (Eds.), Feeding ecology in apes and other primates (pp. 97–122). Cambridge: Cambridge University Press.

[ece34871-bib-0062] Moscovice, L. , Issa, M. , Petrzelkova, K. , Keuler, N. , Snowdon, C. , & Huffman, M. (2007). Fruit availability, chimpanzee diet, and grouping patterns on Rubondo Island, Tanzania. American Journal of Primatology, 69, 487–502. 10.1002/ajp.20350. 17294435

[ece34871-bib-0063] Moynihan, K. J. , Caldwell, E. R. , Sellier, U. L. , Kaul, C. F. , Daetwyler, N. A. , Hayward, G. L. , & Batterhame, G. (2004). Chad Export Project: Environmental Protection Measures. SPE International Conference on Health, Safety, and Environment in Oil and Gas Exploration and Production. Society of Petroleum Engineers.

[ece34871-bib-0064] Murray, C. M. , Eberly, L. E. , & Pusey, A. E. (2006). Foraging strategies as a function of season and rank among wild female chimpanzees (Pan troglodytes). Behavioral Ecology, 17, 1020–1028. 10.1093/beheco/arl042.

[ece34871-bib-0065] Ndimbe, M. S. , Morgan, B. J. , Marino, J. , & Abwe, E. E. (2016). Population density estimate of the Nigeria-Cameroon chimpanzee (Pan troglodytes ellioti) in the Ebo forest. Cameroon: Unpublished report.

[ece34871-bib-0066] Newton‐Fisher, N. E. (1999). The diet of chimpanzees in the Budongo Forest Reserve, Uganda. African Journal of Ecology, 37, 344–354. 10.1046/j.1365-2028.1999.00186.x.

[ece34871-bib-0067] Newton‐Fisher, N. E. (2003). The home range of the Sonso community of chimpanzees from the Budongo Forest, Uganda. African Journal of Ecology, 41, 150–156. 10.1046/j.1365-2028.2003.00408.x.

[ece34871-bib-0068] Nkurunungi, J. B. , Ganas, J. , Robbins, M. M. , & Stanford, C. B. (2004). A comparison of two mountain gorilla habitats in Bwindi Impenetrable National Park, Uganda. African Journal of Ecology, 42, 289–297. 10.1111/j.1365-2028.2004.00523.x.

[ece34871-bib-0069] Oates, J. F. , Bergl, R. , & Linder, J. (2004). Africa's Gulf of Guinea Forests: Biodiversity Patterns and Conservation Priorities. Advances in Applied Biodiversity Science, 6, 1–95.

[ece34871-bib-0070] Ogawa, H. , Idani, G. , Moore, J. , Pintea, L. , & Hernandez‐Aguilar, A. (2007). Sleeping parties and nest distribution of chimpanzees in the savanna woodland, Ugalla, Tanzania. International Journal of Primatology, 28, 1397–1412. 10.1007/s10764-007-9210-0.

[ece34871-bib-0071] Piel, A. K. , Strampelli, P. , Greathead, E. , Hernandez‐Aguilar, R. A. , Moore, J. , & Stewart, F. A. (2017). The diet of open‐habitat chimpanzees (Pan troglodytes schweinfurthii) in the Issa valley, western Tanzania. Journal of Human Evolution, 112, 57–69. 10.1016/j.jhevol.2017.08.016. 29037416

[ece34871-bib-0072] Potts, K. B. , Chapman, C. A. , & Lwanga, J. S. (2009). Floristic heterogeneity between forested sites in Kibale National Park, Uganda: Insights into the fine‐scale determinants of density in a large‐bodied frugivorous primate. Journal of Animal Ecology, 78, 1269–1277. 10.1111/j.1365-2656.2009.01578.x. 19523110

[ece34871-bib-0073] Potts, K. B. , Watts, D. P. , & Wrangham, R. W. (2011). Comparative feeding ecology of two communities of chimpanzees (Pan troglodytes) in Kibale National Park, Uganda. International Journal of Primatology, 32, 669–690. 10.1007/s10764-011-9494-y.

[ece34871-bib-0074] Prado‐Martinez, J. , Sudmant, P. H. , Kidd, J. M. , Li, H. , Kelley, J. L. , Lorente‐Galdos, B. , Veeramah, K. R. , … Marques‐Bonet, T. (2013). Great ape genetic diversity and population history. Nature, 499, 471–475. 10.1038/nature12228. 23823723 PMC3822165

[ece34871-bib-0075] Proctor, J. , Edwards, I. D. , Payton, R. W. , & Nagy, L. (2007). Zonation of forest vegetation and soils of Mount Cameroon, West Africa. Plant Ecology, 192, 251–269. 10.1007/s11258-007-9326-5.

[ece34871-bib-0076] Pruetz, J. D. , & Bertolani, P. (2009). Chimpanzee (Pan troglodytes verus) behavioral responses to stresses associated with living in a savanna‐mosaic environment: Implications for hominin adaptations to open habitats. PaleoAnthropology, 2009, 252–262. 10.4207/PA.2009.ART33.

[ece34871-bib-0077] R Core Team (2017). R: A language and environment for statistical computing. Vienna: R Foundation for Statistical Computing.

[ece34871-bib-0078] Sesink Clee, P. R. , Abwe, E. E. , Ambahe, R. D. , Anthony, N. M. , Fotso, R. , Locatelli, S. , … Gonder, M. K. (2015). Chimpanzee population structure in Cameroon and Nigeria is associated with habitat variation that may be lost under climate change. BMC Evolutionary Biology, 15(1), 2 10.1186/s12862-014-0275-z. 25608567 PMC4314735

[ece34871-bib-0079] Sommer, V. , Adanu, J. , Faucher, I. , & Fowler, A. (2004). Nigerian chimpanzees (Pan troglodytes vellerosus) at Gashaka: Two years of habituation efforts. Folia Primatologica, 75, 295–316. 10.1159/000080208. 15486442

[ece34871-bib-0080] Stanford, C. B. (1998). The social behavior of chimpanzees and bonobos: Empirical evidence and shifting assumptions. Current Anthropology, 39, 399–420. 10.1086/204757.

[ece34871-bib-0081] Stanford, C. B. , & Nkurunungi, J. B. (2003). Behavioral ecology of sympatric chimpanzees and gorillas in Bwindi Impenetrable National Park, Uganda: Diet. International Journal of Primatology, 24, 901–918. 10.1023/A:1024689008159.

[ece34871-bib-0082] Stanford, C. B. , & O’malley, R. C. (2008). Sleeping tree choice by Bwindi chimpanzees. American Journal of Primatology, 70, 642–649. 10.1002/ajp.20539. 18381629

[ece34871-bib-0083] Strindberg, S. , Maisels, F. , Williamson, E. A. , Blake, S. , Stokes, E. J. , Aba’a, R. , … Bakabana, P. C. (2018). Guns, germs, and trees determine density and distribution of gorillas and chimpanzees in Western Equatorial Africa. ScienceAdvances, 4, eaar2964 10.1126/sciadv.aar2964. PMC5916511 29707637

[ece34871-bib-0084] Stumpf, R. (2011). Chimpanzees and bonobos: Inter‐and intraspecies diversity In Campbell C. J., Fuentes A., Mackinnon K. C., Panger M., & Bearder S. K. (Eds.), Primates in perspective (pp. 340–356). Oxford, UK: Oxford University Press.

[ece34871-bib-0085] Sugiyama, Y. (2004). Demographic parameters and life history of chimpanzees at Bossou, Guinea. American Journal of Physical Anthropology, 124, 154–165. 10.1002/ajpa.10345. 15160368

[ece34871-bib-0086] The Plant List (2013). Version 1.1. Published on the Internet; Retrieved from http://www.theplantlist.org/ (accessed 9th June 2018).

[ece34871-bib-0087] Tutin, C. E. G. , Fernandez, M. , Rogers, M. E. , Williamson, E. A. , McGrew, W. C. (1991). Foraging profiles of sympatric lowland gorillas and chimpanzees in the Lope Reserve, Gabon [and Discussion]. Philosophical Transactions of the Royal Society of London. Series B: Biological Sciences, 334, 179–186. 10.1098/rstb.1991.0107. 1685576

[ece34871-bib-0088] Tutin, C. E. , Ham, R. M. , White, L. J. , & Harrison, M. J. (1997). The primate community of the Lope Reserve, Gabon: Diets, responses to fruit scarcity, and effects on biomass. American Journal Primatology, 42, 1–24. 10.1002/(SICI)1098-2345(1997)42:1<1:AID-AJP1>3.0.CO;2-0. 9108968

[ece34871-bib-0089] Tweheyo, M. , & Lye, K. A. (2005). Patterns of frugivory of the Budongo Forest chimpanzees, Uganda. African Journal of Ecology, 43, 282–290. 10.1111/j.1365-2028.2005.00566.x.

[ece34871-bib-0090] van Der Burgt, X. M. , Mackinder, B. A. , Wieringa, J. J. , & De La Estrella, M. (2015). The Gilbertiodendron ogoouense species complex (Leguminosae: Caesalpinioideae), Central Africa. Kew Bulletin, 70, 29 10.1007/s12225-015-9579-4.

[ece34871-bib-0091] van Schaik, C. P. , & Brockman, D. K. (2005). Seasonality in primate ecology, reproduction and life history: An overview In Brockman D. K., & Schaik C. V. (Eds.), Seasonality in primates: Studies of living and extinct human and non‐human primates (pp. 3–20). Cambridge; New York: Cambridge University Press.

[ece34871-bib-0092] Wallis, J. (1995). Seasonal influence on reproduction in chimpanzees of Gombe National Park. International Journal of Primatology, 16, 435–451. 10.1007/BF02735796.

[ece34871-bib-0093] Walsh, P. D. , Abernethy, K. A. , Bermejo, M. , Beyers, R. , De Wachter, P. , Akou, M. E. , … Kilbourn, A. M. (2003). Catastrophic ape decline in western equatorial Africa. Nature, 422, 611–614. 10.1038/nature01566. 12679788

[ece34871-bib-0094] Watts, D. P. , & Mitani, J. C. (2001). Boundary patrols and intergroup encounters in wild chimpanzees. Behaviour, 138, 299–327. 10.1163/15685390152032488.

[ece34871-bib-0095] Watts, D. P. , Potts, K. B. , Lwanga, J. S. , & Mitani, J. C. (2012). Diet of chimpanzees (Pan troglodytes schweinfurthii) at Ngogo, Kibale National Park, Uganda, 1. Diet composition and diversity. American Journal of Primatology, 74, 114–129. 10.1002/ajp.21016. 22109938

[ece34871-bib-0096] White, L. J. (1994). Patterns of fruit‐fall phenology in the Lopé Reserve, Gabon. Journal of Tropical Ecology, 10, 289–312. 10.1017/S0266467400007975.

[ece34871-bib-0097] Whiten, A. , Goodall, J. , Mcgrew, W. C. , Nishida, T. , Reynolds, V. , Sugiyama, Y. , … Boesch, C. (1999). Cultures in chimpanzees. Nature, 399, 682–685. 10.1038/21415. 10385119

[ece34871-bib-0098] Willie, J. , Tagg, N. , Petre, C.‐A. , Pereboom, Z. , & Lens, L. (2014). Plant selection for nest building by western lowland gorillas in Cameroon. Primates, 55, 41–49. 10.1007/s10329-013-0363-5. 23732768

[ece34871-bib-0099] Worman, C. O. D. , & Chapman, C. A. (2006). Densities of two frugivorous primates with respect to forest and fragment tree species composition and fruit availability. International Journal of Primatology, 27, 203–225. 10.1007/s10764-005-9007-y.

[ece34871-bib-0100] Wrangham, R. W. , Chapman, C. A. , Clark‐Arcadi, A. P. , & Isabirye‐Basuta, G. (1996). Social ecology of Kanyawara chimpanzees: Implications for understanding the costs of great ape groups In Mcgrew W., Marchant L., & Nishida T. (Eds.), Great ape societies (pp. 45–57). Cambridge: Cambridge University Press.

[ece34871-bib-0101] Wrangham, R. W. , & Smuts, B. B. (1980). Sex differences in the behavioural ecology of chimpanzees in the Gombe National Park, Tanzania. Journal of Reproduction and Fertility. (Suppl 28), 13–31. 6934308

[ece34871-bib-0102] Wrangham, R. , Conklin, N. , Chapman, C. , Hunt, K. , Milton, K. , Rogers, E. , … Barton, R. (1991). The significance of fibrous foods for Kibale Forest chimpanzees [and Discussion]. Philosophical Transactions of the Royal Society B: Biological Sciences, 334, 171–178. 10.1098/rstb.1991.0106. 1685575

[ece34871-bib-0103] Yamakoshi, G. (1998). Dietary responses to fruit scarcity of wild chimpanzees at Bossou, Guinea: Possible implications for ecological importance of tool use. American Journal of Physical Anthropology, 106, 283–295. 10.1002/(SICI)1096-8644(199807)106:3<283:AID-AJPA2>3.0.CO;2-O. 9696145

[ece34871-bib-0104] Yamakoshi, G. (2004). Food seasonality and socioecology in Pan: Are West African chimpanzees another bonobo? African Study Monographs, 25, 45–60. 10.14989/68227.

