# Supplementary material for: Habitat differentiation among three Nigeria–Cameroon chimpanzee (Pan troglodytes ellioti) populations
Source: Ecol Evol. 2019 Jan 10;9(3):1489–500. doi: 10.1002/ece3.4871 (PMC6374666; doi:10.1002/ece3.4871)
Supplement: Supplementary file 1 [file ECE3-9-1489-s001.docx]

**Figure S1.** Annual precipitation at the three study sites.

**Table S1.** Plant species list (≥10 cm DBH), number of stems per species and family for human-modified rainforest-Bekob.

| **Species** | **Count** | **Family** |
| --- | --- | --- |
| *Thomandersia sp.* | 8 | Acanthaceae |
| *Dasylepis racemosa* | 1 | Achariaceae |
| *Scottellia coriacea* | 3 | Achariaceae |
| *Antrocaryon klaineanum* | 3 | Anacardiaceae |
| *Lannea welwitschii* | 17 | Anacardiaceae |
| *Pseudospondias microcarpa* | 28 | Anacardiaceae |
| *Trichoscypha acuminata* | 4 | Anacardiaceae |
| *Trichoscypha sp.* | 88 | Anacardiaceae |
| *Sorindeia sp.* | 1 | Anacardiaceae |
| *Anisophyllea sp.* | 55 | Anisophylleaceae |
| *Annickia chlorantha* | 8 | Annonaceae |
| *Anonidium mannii* | 1 | Annonaceae |
| *Piptostigma fuscum* | 6 | Annonaceae |
| *Piptostigma sp.* | 21 | Annonaceae |
| *Uvariodendron connivens* | 3 | Annonaceae |
| *Uvariopsis sp.* | 1 | Annonaceae |
| *Xylopia aethiopica* | 10 | Annonaceae |
| *Cleistopholis glauca* | 5 | Annonaceae |
| *Cleistopholis sp.* | 1 | Annonaceae |
| *Greenwayodendron suaveolens* | 9 | Annonaceae |
| *Monodora myristica* | 1 | Annonaceae |
| *Monodora sp.* | 30 | Annonaceae |
| *Xylopia africana* | 5 | Annonaceae |
| *Xylopia phliocodora* | 1 | Annonaceae |
| *Xylopia sp.* | 16 | Annonaceae |
| *Alstonia boonei* | 7 | Apocynaceae |
| *Picralima nitida* | 39 | Apocynaceae |
| *Tabernaemontana crassa* | 476 | Apocynaceae |
| *Funtumia elastica* | 11 | Apocynaceae |
| *Rauvolfia macrophylla* | 21 | Apocynaceae |
| *Rauvolfia sp.* | 1 | Apocynaceae |
| *Rauvolfia vomitoria* | 27 | Apocynaceae |
| *Elaeis guineensis* | 33 | Arecaceae |
| *Raphia regalis* | 3 | Arecaceae |
| *Raphia sp.* | 6 | Arecaceae |
| *Dracaena cerasifera* | 6 | Asparagaceae |
| *Dracaena sp.* | 1 | Asparagaceae |
| *Kigelia africana* | 1 | Bignoniaceae |
| *Markhamia tomentosa* | 2 | Bignoniaceae |
| *Spathodea campanulata* | 1 | Bignoniaceae |
| *Cordia sp.* | 1 | Boraginaceae |
| *Cordia platythyrsa* | 8 | Boraginaceae |
| *Hoplestigma cf. pierreanum* | 1 | Boraginaceae |
| *Hoplestigma sp.* | 1 | Boraginaceae |
| *Dacryodes sp.* | 39 | Burseraceae |
| *Canarium schweinfurthii* | 3 | Burseraceae |
| *Santiria trimera* | 84 | Burseraceae |
| *Mammea africana* | 25 | Calophyllaceae |
| *Euadenia trifoliolata* | 2 | Capparaceae |
| *Ritchiea erecta* | 3 | Capparaceae |
| *Magnistipula sp.* | 3 | Chrysobalanaceae |
| *Maranthes sp.* | 3 | Chrysobalanaceae |
| *Parinari excelsa* | 2 | Chrysobalanaceae |
| *Allanblackia floribunda* | 2 | Clusiaceae |
| *Allanblackia gabonensis* | 10 | Clusiaceae |
| *Allanblackia sp.* | 6 | Clusiaceae |
| *Symphonia globulifera* | 13 | Clusiaceae |
| *Vismia guineensis* | 1 | Clusiaceae |
| *Vismia sp.* | 1 | Clusiaceae |
| *Garcinia conrauana* | 185 | Clusiaceae |
| *Garcinia lucida* | 5 | Clusiaceae |
| *Garcinia mannii* | 16 | Clusiaceae |
| *Garcinia ovalifolia* | 8 | Clusiaceae |
| *Garcinia smeathmannii* | 32 | Clusiaceae |
| *Garcinia sp.* | 30 | Clusiaceae |
| *Terminalia superba* | 9 | Combretaceae |
| *Cyathea camerooniana* | 1 | Cyatheaceae |
| *Tapura africana* | 13 | Dichapetalaceae |
| *Tapura sp.* | 4 | Dichapetalaceae |
| *Diospyros hoyleana* | 3 | Ebenaceae |
| *Diospyros sp.* | 68 | Ebenaceae |
| *Anthostema aubryanum* | 1 | Euphorbiaceae |
| *Croton oligandrus* | 7 | Euphorbiaceae |
| *Cyrtogonone argentea* | 5 | Euphorbiaceae |
| *Dichostemma glaucescens* | 4 | Euphorbiaceae |
| *Discoglypremna caloneura* | 9 | Euphorbiaceae |
| *Elaeophorbia drupifera* | 1 | Euphorbiaceae |
| *Macaranga barteri* | 4 | Euphorbiaceae |
| *Macaranga lunifolia* | 41 | Euphorbiaceae |
| *Macaranga sp.* | 9 | Euphorbiaceae |
| *Mallotus oppositifolius* | 3 | Euphorbiaceae |
| *Mareyopsis longifolia* | 16 | Euphorbiaceae |
| *Plagiostyles africana* | 1 | Euphorbiaceae |
| *Pseudagrostistachys africana* | 1 | Euphorbiaceae |
| *Mareyopsis sp.* | 1 | Euphorbiaceae |
| *Neoboutonia laevis* | 1 | Euphorbiaceae |
| *Neoboutonia mannii* | 11 | Euphorbiaceae |
| *Ricinodendron heudelotii* | 3 | Euphorbiaceae |
| *Sapium cuneatum* | 1 | Euphorbiaceae |
| *Shirakiopsis elliptica* | 6 | Euphorbiaceae |
| *Anthocleista nobilis* | 1 | Gentianaceae |
| *Anthocleista schweinfurthii* | 1 | Gentianaceae |
| *Afrostyrax lepidophyllus* | 16 | Huaceae |
| *Afrostyrax sp.* | 10 | Huaceae |
| *Harungana madagascariensis* | 5 | Hypericaceae |
| *Desbordesia glaucescens* | 4 | Irvingiaceae |
| *Irvingia gabonensis* | 2 | Irvingiaceae |
| *Klainedoxa gabonensis* | 14 | Irvingiaceae |
| *Klainedoxa sp.* | 2 | Irvingiaceae |
| *Klainedoxa trillesii* | 4 | Irvingiaceae |
| *Vitex grandifolia* | 138 | Lamiaceae |
| *Vitex sp.* | 2 | Lamiaceae |
| *Hypodaphnis zenkeri* | 13 | Lauraceae |
| *Beilschmiedia mannii* | 1 | Lauraceae |
| *Beilschmiedia sp.* | 47 | Lauraceae |
| *Crateranthus cameroonensis* | 38 | Lecythidaceae |
| *Petersianthus macrocarpus* | 4 | Lecythidaceae |
| *Rhaptopetalum sessilifolium* | 1 | Lecythidaceae |
| *Rhaptopetalum sp.* | 2 | Lecythidaceae |
| *Afzelia bipindensis* | 1 | Leguminosae |
| *Afzelia sp.* | 1 | Leguminosae |
| *Albizia adianthifolia* | 9 | Leguminosae |
| *Albizia sp.* | 1 | Leguminosae |
| *Albizia zygia* | 5 | Leguminosae |
| *Amphimas ferrugineus* | 1 | Leguminosae |
| *Amphimas pterocarpoides* | 2 | Leguminosae |
| *Amphimas sp.* | 3 | Leguminosae |
| *Anthonotha ferruginea* | 20 | Leguminosae |
| *Anthonotha sp.* | 9 | Leguminosae |
| *Cynometra hankei* | 1 | Leguminosae |
| *Dialium pachyphyllum* | 2 | Leguminosae |
| *Dialium sp.* | 13 | Leguminosae |
| *Hymenostegia afzelii* | 96 | Leguminosae |
| *Hymenostegia cf. brachyura* | 1 | Leguminosae |
| *Hymenostegia sp.* | 4 | Leguminosae |
| *Leonardoxa (false)* | 1 | Leguminosae |
| *Leonardoxa africana* | 53 | Leguminosae |
| *Leosenera talbotii* | 1 | Leguminosae |
| *Pentaclethra macrophylla* | 1 | Leguminosae |
| *Piptadeniatrum africanum* | 1 | Leguminosae |
| *Plagiosiphon emarginatus* | 3 | Leguminosae |
| *Plagiosiphon longitubus* | 3 | Leguminosae |
| *Plagiosiphon multijugus* | 1 | Leguminosae |
| *Plagiosiphon sp.* | 22 | Leguminosae |
| *Prioria joveri* | 3 | Leguminosae |
| *Prioria/Oxystigma?* | 1 | Leguminosae |
| *Pterocarpus soyauxii* | 2 | Leguminosae |
| *Tetrapleura tetraptera* | 2 | Leguminosae |
| *Baphia sp.* | 9 | Leguminosae |
| *Hylodendron gabunense* | 15 | Leguminosae |
| *Hylodendron sp.* | 2 | Leguminosae |
| *Millettia sp.* | 1 | Leguminosae |
| *Oddoniodendron sp.* | 1 | Leguminosae |
| *Oxystigma/Gossuselerodendron* | 1 | Leguminosae |
| *Stemonocoleus micranthus* | 1 | Leguminosae |
| *Zenkerella citrina* | 84 | Leguminosae |
| *Lepidobotrys staudtii* | 5 | Lepidobotryaceae |
| *Strychnos sp.* | 6 | Loganiaceae |
| *Strychnos staudtii* | 1 | Loganiaceae |
| *Desplatsia dewevrei* | 4 | Malvaceae |
| *Desplatsia sp.* | 10 | Malvaceae |
| *Duboscia sp.* | 1 | Malvaceae |
| *Leptonychia sp.* | 4 | Malvaceae |
| *Ceiba pentandra* | 1 | Malvaceae |
| *Cola acuminata* | 12 | Malvaceae |
| *Cola cauliflora* | 6 | Malvaceae |
| *Cola lateritia* | 9 | Malvaceae |
| *Cola lepidota* | 5 | Malvaceae |
| *Cola nitida* | 5 | Malvaceae |
| *Cola pachycarpa* | 1 | Malvaceae |
| *Cola rostrata* | 5 | Malvaceae |
| *Cola sp.* | 230 | Malvaceae |
| *Cola verticillata* | 26 | Malvaceae |
| *Grewia coriacea* | 40 | Malvaceae |
| *Grewia sp.* | 7 | Malvaceae |
| *Sterculia oblonga* | 1 | Malvaceae |
| *Sterculia tragacantha* | 8 | Malvaceae |
| *Warneckea sp.* | 2 | Melastomataceae |
| *Lovoa trichilioides* | 5 | Meliaceae |
| *Trichilia prieuriana* | 10 | Meliaceae |
| *Trichilia rubescens* | 54 | Meliaceae |
| *Trichilia sp.* | 40 | Meliaceae |
| *Carapa dinklagei* | 5 | Meliaceae |
| *Carapa sp.* | 40 | Meliaceae |
| *Entandrophragma sp.* | 1 | Meliaceae |
| *Guarea mayombensis* | 7 | Meliaceae |
| *Guarea sp.* | 16 | Meliaceae |
| *Guarea thompsonii* | 3 | Meliaceae |
| *Bersama sp.* | 1 | Melianthaceae |
| *Antiaris africana* | 1 | Moraceae |
| *Antiaris sp.* | 1 | Moraceae |
| *Treculia obovoidea* | 9 | Moraceae |
| *Trilepisium madagascariense* | 3 | Moraceae |
| *Ficus sp.* | 12 | Moraceae |
| *Milicia excelsa* | 17 | Moraceae |
| *Pycnanthus angolensis* | 112 | Myristicaceae |
| *Coelocaryon preussii* | 30 | Myristicaceae |
| *Scyphocephalium mannii* | 12 | Myristicaceae |
| *Staudtia kamerunensis* | 1 | Myristicaceae |
| *Psidium guajava* | 2 | Myrtaceae |
| *Syzygium guineensis* | 3 | Myrtaceae |
| *Syzygium sp.* | 9 | Myrtaceae |
| *Eugenia sp.* | 3 | Myrtaceae |
| *Lophira alata* | 14 | Ochnaceae |
| *Campylospermum calanthum* | 2 | Ochnaceae |
| *Ochna calodendron* | 10 | Ochnaceae |
| *Ochna sp.* | 1 | Ochnaceae |
| *Rhabdophyllum sp.* | 8 | Ochnaceae |
| *Aptandra sp.* | 1 | Olacaceae |
| *Coula edulis* | 5 | Olacaceae |
| *Diogoa zenkeri* | 80 | Olacaceae |
| *Heisteria parviflora* | 3 | Olacaceae |
| *Heisteria sp.* | 8 | Olacaceae |
| *Octoknema affinis* | 26 | Olacaceae |
| *Octoknema sp.* | 16 | Olacaceae |
| *Ongokea gore* | 2 | Olacaceae |
| *Strombosia grandifolia* | 166 | Olacaceae |
| *Strombosia pustulata* | 4 | Olacaceae |
| *Strombosia scheffleri* | 22 | Olacaceae |
| *Strombosia sp.* | 142 | Olacaceae |
| *Strombosiopsis tetrandra* | 15 | Olacaceae |
| *Barteria fistulosa* | 1 | Passifloraceae |
| *Medusandra mpomiana* | 22 | Peridiscaceae |
| *Antidesma laciniatum* | 2 | Phyllanthaceae |
| *Antidesma membranaceum* | 51 | Phyllanthaceae |
| *Antidesma sp.* | 18 | Phyllanthaceae |
| *Maesobotrya barteri* | 22 | Phyllanthaceae |
| *Maesobotrya sp.* | 33 | Phyllanthaceae |
| *Uapaca guineensis* | 155 | Phyllanthaceae |
| *Uapaca sp.* | 10 | Phyllanthaceae |
| *Bridelia micrantha* | 5 | Phyllanthaceae |
| *Bridelia sp.* | 1 | Phyllanthaceae |
| *Hymenocardia sp.* | 6 | Phyllanthaceae |
| *Hymenocardia ulmoides* | 2 | Phyllanthaceae |
| *Margaritaria discoidea* | 94 | Phyllanthaceae |
| *Oldfieldia sp.* | 1 | Picrodendraceae |
| *Pittosporum or Peddea* | 2 | Pittospaceae |
| *Drypetes aframensis* | 5 | Putranjivaceae |
| *Drypetes aylmeri* | 5 | Putranjivaceae |
| *Drypetes principum* | 19 | Putranjivaceae |
| *Drypetes sp.* | 297 | Putranjivaceae |
| *Lasiodiscus mannii* | 2 | Rhamnaceae |
| *Lasiodiscus sp.* | 1 | Rhamnaceae |
| *Maesopsis eminii* | 7 | Rhamnaceae |
| *Cassipourea sp.* | 2 | Rhizophoraceae |
| *Cuviera longiflora* | 14 | Rubiaceae |
| *Dictyandra sp.* | 1 | Rubiaceae |
| *Ixora sp.* | 2 | Rubiaceae |
| *Pauridiantha efferata* | 14 | Rubiaceae |
| *Pauridiantha efulensis* | 2 | Rubiaceae |
| *Pauridiantha sp.* | 2 | Rubiaceae |
| *Pausinystalia macroceras* | 5 | Rubiaceae |
| *Pausinystalia sp.* | 1 | Rubiaceae |
| *Porterandia cladantha* | 2 | Rubiaceae |
| *Psychotria camptopus* | 1 | Rubiaceae |
| *Psychotria sp.* | 1 | Rubiaceae |
| *Psychotria venosa* | 13 | Rubiaceae |
| *Psydrax arnoldiana* | 1 | Rubiaceae |
| *Psydrax sp.* | 4 | Rubiaceae |
| *Tricalysia sp.* | 35 | Rubiaceae |
| *Aulacocalyx sp.* | 5 | Rubiaceae |
| *Coffea sp.* | 1 | Rubiaceae |
| *Heinsia crinita* | 3 | Rubiaceae |
| *Massularia acuminata* | 3 | Rubiaceae |
| *Morinda lucida* | 3 | Rubiaceae |
| *Nauclea diderrichii* | 4 | Rubiaceae |
| *Nauclea pobeguinii* | 5 | Rubiaceae |
| *Rothmannia sp.* | 8 | Rubiaceae |
| *Schumanniophyton magnificum* | 19 | Rubiaceae |
| *Afraegle paniculata* | 1 | Rutaceae |
| *Vepris sp.* | 2 | Rutaceae |
| *Vepris suaveolens* | 1 | Rutaceae |
| *Vepris trifoliolata* | 6 | Rutaceae |
| *Clausena anisata* | 4 | Rutaceae |
| *Zanthoxylum sp.* | 28 | Rutaceae |
| *Zanthoxylum tessmannii* | 1 | Rutaceae |
| *Dovyalis zenkeri* | 4 | Salicaceae |
| *Homalium letestui* | 11 | Salicaceae |
| *Homalium sp.* | 1 | Salicaceae |
| *Oncoba blackii* | 39 | Salicaceae |
| *Oncoba calodendron* | 2 | Salicaceae |
| *Oncoba dentata* | 7 | Salicaceae |
| *Oncoba glauca* | 45 | Salicaceae |
| *Oncoba sp.* | 5 | Salicaceae |
| *Oncoba welwitschii* | 164 | Salicaceae |
| *Allophylus africanus* | 4 | Sapindaceae |
| *Allophylus sp.* | 4 | Sapindaceae |
| *Deinbollia sp.* | 2 | Sapindaceae |
| *Lychnodiscus sp.* | 2 | Sapindaceae |
| *Placodiscus sp.* | 2 | Sapindaceae |
| *Blighia sp.* | 9 | Sapindaceae |
| *Chytranthus sp.* | 3 | Sapindaceae |
| *Eriocoelum macrocarpum* | 8 | Sapindaceae |
| *Eriocoelum sp.* | 3 | Sapindaceae |
| *Pouteria sp.* | 5 | Sapotaceae |
| *Synsepalum msolo* | 4 | Sapotaceae |
| *Synsepalum sp.* | 3 | Sapotaceae |
| *Englerophytum stelechantha* | 2 | Sapotaceae |
| *Gambeya sp.* | 3 | Sapotaceae |
| *Omphalocarpum sp.* | 1 | Sapotaceae |
| *Quassia sanguinea* | 5 | Simaroubaceae |
| *Dicranolepis sp.* | 4 | Thymelaeaceae |
| *Liana* | 70 | Undefined |
| *Unknown* | 405 | Undefined |
| *Musanga cecropioides* | 9 | Urticaceae |
| *Myrianthus arboreus* | 1 | Urticaceae |
| *Myrianthus serratus* | 5 | Urticaceae |
| *Myrianthus sp.* | 1 | Urticaceae |
| *Rinorea oblongifolia* | 20 | Violaceae |
| *Rinorea sp.* | 12 | Violaceae |
| *Balanites sp.* | 1 | Zygophyllaceae |

**Table S2.** Plant species list (≥10 cm DBH), number of stems per species and family for rainforest-Njuma

| **Species** | **Count** | **Family** |
| --- | --- | --- |
| *Thomandersia sp.* | 4 | Acanthaceae |
| *Dasylepis racemosa* | 16 | Achariaceae |
| *Scottellia coriacea* | 3 | Achariaceae |
| *Lannea welwitschii* | 4 | Anacardiaceae |
| *Pseudospondias microcarpa* | 2 | Anacardiaceae |
| *Sorindeia grandifolia* | 1 | Anacardiaceae |
| *Sorindeia sp.* | 2 | Anacardiaceae |
| *Trichoscypha acuminata* | 2 | Anacardiaceae |
| *Trichoscypha sp.* | 26 | Anacardiaceae |
| *Anisophyllea polyneura* | 2 | Anisophylleaceae |
| *Anisophyllea sp.* | 139 | Anisophylleaceae |
| *Poga oleosa* | 1 | Anisophylleaceae |
| *Poga sp.* | 2 | Anisophylleaceae |
| *Annickia chlorantha* | 23 | Annonaceae |
| *Cleistopholis glauca* | 13 | Annonaceae |
| *Cleistopholis patens* | 6 | Annonaceae |
| *Duguetia staudtii* | 5 | Annonaceae |
| *Greenwayodendron sp.* | 1 | Annonaceae |
| *Greenwayodendron suaveolens* | 25 | Annonaceae |
| *Hexalobus crispiflorus* | 1 | Annonaceae |
| *Hexalobus sp.* | 1 | Annonaceae |
| *Isolona sp.* | 2 | Annonaceae |
| *Monodora myristica* | 3 | Annonaceae |
| *Monodora sp.* | 8 | Annonaceae |
| *Piptostigma sp.* | 5 | Annonaceae |
| *Uvariodendron connivens* | 1 | Annonaceae |
| *Uvariodendron giganteum* | 1 | Annonaceae |
| *Uvariodendron sp.* | 6 | Annonaceae |
| *Uvariopsis congolana* | 1 | Annonaceae |
| *Uvariopsis sp.* | 4 | Annonaceae |
| *Xylopia aethiopica* | 2 | Annonaceae |
| *Xylopia rubescens* | 2 | Annonaceae |
| *Xylopia sp.* | 7 | Annonaceae |
| *Xylopia staudtii* | 5 | Annonaceae |
| *Alstonia boonei* | 24 | Apocynaceae |
| *Funtumia elastica* | 2 | Apocynaceae |
| *Picralima nitida* | 15 | Apocynaceae |
| *Rauvolfia macrophylla* | 10 | Apocynaceae |
| *Rauvolfia sp.* | 1 | Apocynaceae |
| *Rauvolfia vomitoria* | 4 | Apocynaceae |
| *Tabernaemontana* | 1 | Apocynaceae |
| *Tabernaemontana contorta* | 3 | Apocynaceae |
| *Tabernaemontana crassa* | 110 | Apocynaceae |
| *Elaeis guineensis* | 2 | Arecaceae |
| *Raphia sp.* | 3 | Arecaceae |
| *Newbouldia laevis* | 1 | Bignoniaceae |
| *Spathodea campanulata* | 1 | Bignoniaceae |
| *Canarium schweinfurthii* | 1 | Burseraceae |
| *Dacryodes macrophylla* | 2 | Burseraceae |
| *Dacryodes sp.* | 37 | Burseraceae |
| *Santiria trimera* | 43 | Burseraceae |
| *Ritchiea erecta* | 2 | Capparaceae |
| *Leptaulus sp.* | 5 | Cardiopteridaceae |
| *Magnistipula sp.* | 2 | Chrysobalanaceae |
| *Maranthes sp.* | 2 | Chrysobalanaceae |
| *Parinari excelsa* | 2 | Chrysobalanaceae |
| *Allanblackia sp.* | 3 | Clusiaceae |
| *Garcinia conrauana* | 32 | Clusiaceae |
| *Garcinia kola* | 1 | Clusiaceae |
| *Garcinia lucida* | 25 | Clusiaceae |
| *Garcinia mannii* | 28 | Clusiaceae |
| *Garcinia ovalifolia* | 7 | Clusiaceae |
| *Garcinia smeathmannii* | 26 | Clusiaceae |
| *Garcinia sp.* | 78 | Clusiaceae |
| *Garcinia thompsonii* | 1 | Clusiaceae |
| *Pentadesma sp.* | 4 | Clusiaceae |
| *Pentadesma grandifolia* | 1 | Clusiaceae |
| *Symphonia globulifera* | 9 | Clusiaceae |
| *Terminalia superba* | 16 | Combretaceae |
| *Santaloidella giletii* | 1 | Connaraceae |
| *Calycobolus africana* | 2 | Convolvulaceae |
| *Neuropeltis sp.* | 3 | Convolvulaceae |
| *Tetracera alnifolia* | 3 | Dilleniaceae |
| *Diospyros bipidensis* | 168 | Ebenaceae |
| *Diospyros cinnabarina* | 4 | Ebenaceae |
| *Diospyros exprudensis* | 1 | Ebenaceae |
| *Diospyros hoyleana* | 3 | Ebenaceae |
| *Diospyros longifolia* | 1 | Ebenaceae |
| *Diospyros preussii* | 1 | Ebenaceae |
| *Diospyros sp.* | 338 | Ebenaceae |
| *Diospyros suaveolens* | 10 | Ebenaceae |
| *Cavacoa quintassi* | 1 | Euphorbiaceae |
| *Croton oligandrus* | 7 | Euphorbiaceae |
| *Cyrtogonone argentea* | 7 | Euphorbiaceae |
| *Dichostemma glaucescens* | 55 | Euphorbiaceae |
| *Discoglypremna caloneura* | 2 | Euphorbiaceae |
| *Faux Mareyopsis* | 1 | Euphorbiaceae |
| *Grossera sp.* | 4 | Euphorbiaceae |
| *Grossera vignei* | 4 | Euphorbiaceae |
| *Macaranga lunifolia* | 9 | Euphorbiaceae |
| *Maesobotrya barteri* | 3 | Euphorbiaceae |
| *Maesobotrya dugetii* | 1 | Euphorbiaceae |
| *Maesobotrya sp.* | 1 | Euphorbiaceae |
| *Maprounea africana* | 1 | Euphorbiaceae |
| *Mareyopsis longifolia* | 63 | Euphorbiaceae |
| *Mareyopsis sp.* | 7 | Euphorbiaceae |
| *Neoboutonia mannii* | 3 | Euphorbiaceae |
| *Plagiostyles africana* | 2 | Euphorbiaceae |
| *Ricinodendron heudelotii* | 5 | Euphorbiaceae |
| *Anthocleista nobilis* | 4 | Gentianaceae |
| *Anthocleista vogelli* | 1 | Gentianaceae |
| *Desbordesia glaucescens* | 92 | Irvingiaceae |
| *Irvingia gabonensis* | 36 | Irvingiaceae |
| *Irvingia grandifolia* | 4 | Irvingiaceae |
| *Irvingia sp.* | 3 | Irvingiaceae |
| *Klainedoxa gabonensis* | 16 | Irvingiaceae |
| *Klainedoxa trillesii* | 7 | Irvingiaceae |
| *Vitex grandifolia* | 36 | Lamiaceae |
| *Beilschmiedia mannii* | 1 | Lauraceae |
| *Beilschmiedia obscura* | 1 | Lauraceae |
| *Beilschmiedia sp.* | 21 | Lauraceae |
| *Hypodaphnis zenkeri* | 23 | Lauraceae |
| *Crateranthus sp.* | 1 | Lecythidaceae |
| *Napolaeona egertonii* | 2 | Lecythidaceae |
| *Napolaeona sp.* | 7 | Lecythidaceae |
| *Oubanguia sp.* | 3 | Lecythidaceae |
| *Petersianthus macrocarpus* | 3 | Lecythidaceae |
| *Afzelia bipindensis* | 2 | Leguminosae |
| *Afzelia sp.* | 2 | Leguminosae |
| *Albizia adianthifolia* | 1 | Leguminosae |
| *Anthonotha lamprophylla* | 5 | Leguminosae |
| *Anthonotha macrophylla* | 2 | Leguminosae |
| *Anthonotha schweinfurthii* | 1 | Leguminosae |
| *Anthonotha sp.* | 9 | Leguminosae |
| *Baphia sp.* | 5 | Leguminosae |
| *Berlinia bracteosa* | 13 | Leguminosae |
| *Berlinia grandifolia* | 2 | Leguminosae |
| *Berlinia sp.* | 14 | Leguminosae |
| *Bikinia letestui* | 3 | Leguminosae |
| *Bikinia sp.* | 1 | Leguminosae |
| *Brachystegia cynometroides* | 33 | Leguminosae |
| *Brachystegia mildbraedii* | 1 | Leguminosae |
| *Calpocalux sp.* | 1 | Leguminosae |
| *Calpocalyx dinklagei* | 2 | Leguminosae |
| *Cryptosepalum sp.* | 7 | Leguminosae |
| *Cylicodiscus gabunensis* | 3 | Leguminosae |
| *Cynometra hankei* | 15 | Leguminosae |
| *Dialium dinklagei* | 1 | Leguminosae |
| *Dialium pachyphyllum* | 4 | Leguminosae |
| *Dialium sp.* | 23 | Leguminosae |
| *Distemonanthus benthamianus* | 5 | Leguminosae |
| *Erythrina mildbraedii* | 1 | Leguminosae |
| *Gilbertiodendron brachystegioides* | 1 | Leguminosae |
| *Gilbertiodendron ebo* | 7 | Leguminosae |
| *Gilbertiodendron sp.* | 2 | Leguminosae |
| *Hylodendron gabunense* | 23 | Leguminosae |
| *Hymenostegia afzelii* | 19 | Leguminosae |
| *Hymenostegia sp.* | 1 | Leguminosae |
| *Julbernardia pellegriniana* | 4 | Leguminosae |
| *Julbernardia seretii* | 1 | Leguminosae |
| *Leonardoxa africana* | 9 | Leguminosae |
| *Loesenera talbotii* | 3 | Leguminosae |
| *Microberlinia bisulcata* | 17 | Leguminosae |
| *Milletia sp.* | 3 | Leguminosae |
| *Newtonia sp.* | 1 | Leguminosae |
| *Parkia bicolor* | 1 | Leguminosae |
| *Pentaclethra macrophylla* | 7 | Leguminosae |
| *Piptadeniastrum africanum* | 10 | Leguminosae |
| *Plagiosiphon (faux)* | 1 | Leguminosae |
| *Plagiosiphon emarginatus* | 10 | Leguminosae |
| *Plagiosiphon longitubus* | 5 | Leguminosae |
| *Plagiosiphon sp.* | 14 | Leguminosae |
| *Prioria balsamifirum* | 1 | Leguminosae |
| *Pterocarpus mildbraedii* | 7 | Leguminosae |
| *Pterocarpus soyauxii* | 2 | Leguminosae |
| *Zenkerella citrina* | 32 | Leguminosae |
| *Lepidobotrys staudtii* | 6 | Lepidobotryaceae |
| *Strychnos sp.* | 22 | Loganiceae |
| *Strychnos staudtii* | 5 | Loganiceae |
| *Cola argentea* | 1 | Malvaceae |
| *Cola chlamydantha* | 4 | Malvaceae |
| *Cola lateritia* | 8 | Malvaceae |
| *Cola lepidota* | 3 | Malvaceae |
| *Cola nitida* | 7 | Malvaceae |
| *Cola rostrata* | 11 | Malvaceae |
| *Cola sp.* | 131 | Malvaceae |
| *Cola verticillata* | 7 | Malvaceae |
| *Duboscia macrocarpa* | 3 | Malvaceae |
| *Grewia coriacea* | 30 | Malvaceae |
| *Grewia sp.* | 4 | Malvaceae |
| *Sterculia sp.* | 1 | Malvaceae |
| *Sterculia tragacantha* | 4 | Malvaceae |
| *Carapa dinklagei* | 24 | Meliaceae |
| *Carapa sp.* | 29 | Meliaceae |
| *Entandophragma utile* | 1 | Meliaceae |
| *Guarea mayombensis* | 2 | Meliaceae |
| *Guarea sp.* | 8 | Meliaceae |
| *Guarea thompsonii* | 3 | Meliaceae |
| *Trichilia prieuriana* | 1 | Meliaceae |
| *Trichilia rubescens* | 18 | Meliaceae |
| *Trichilia sp.* | 20 | Meliaceae |
| *Trichilia welwitschi* | 1 | Meliaceae |
| *Antiaris africana* | 2 | Moraceae |
| *Ficus sp.* | 6 | Moraceae |
| *Ficus sur* | 2 | Moraceae |
| *Milicia excelsa* | 1 | Moraceae |
| *Treculia africana* | 2 | Moraceae |
| *Treculia obovoidea* | 7 | Moraceae |
| *Treculia sp.* | 22 | Moraceae |
| *Coelocaryon preussii* | 41 | Myristicaceae |
| *Coelocaryon sp.* | 1 | Myristicaceae |
| *Pycnanthus angolensis* | 87 | Myristicaceae |
| *Scyphocephalium mannii* | 71 | Myristicaceae |
| *Staudtia kamerunensis* | 57 | Myristicaceae |
| *Eugenia sp.* | 2 | Myrtaceae |
| *Syzygium guineensis* | 2 | Myrtaceae |
| *Syzygium sp.* | 10 | Myrtaceae |
| *Campylospermum sp.* | 1 | Ochnaceae |
| *Faux Ochna calodendron* | 1 | Ochnaceae |
| *Faux Rhabdophyllum* | 1 | Ochnaceae |
| *Lophira alata* | 27 | Ochnaceae |
| *Ochna sp.* | 2 | Ochnaceae |
| *Rhabdophyllum sp* | 53 | Ochnaceae |
| *Coula edulis* | 59 | Olacaceae |
| *Diogoa zenkeri* | 298 | Olacaceae |
| *Faux coula* | 1 | Olacaceae |
| *Heisteria parviflora* | 1 | Olacaceae |
| *Heisteria sp.* | 39 | Olacaceae |
| *Heisteria trillesiana* | 6 | Olacaceae |
| *Octoknema affinis* | 18 | Olacaceae |
| *Octoknema sp.* | 2 | Olacaceae |
| *Ongokea gore* | 7 | Olacaceae |
| *Strombosia grandifolia* | 115 | Olacaceae |
| *Strombosia pustulata* | 64 | Olacaceae |
| *Strombosia scheffleri* | 35 | Olacaceae |
| *Strombosia sp.* | 200 | Olacaceae |
| *Strombosiopsis tetrandra* | 39 | Olacaceae |
| *Panda oleosa* | 10 | Pandaceae |
| *Barteria fistulosa* | 3 | Passifloraceae |
| *Barteria sp.* | 1 | Passifloraceae |
| *Medusandra mpomiana* | 1 | Peridiscaceae |
| *Antidesma laciniatum* | 2 | Phyllanthaceae |
| *Antidesma membranaceum* | 13 | Phyllanthaceae |
| *Antidesma sp.* | 27 | Phyllanthaceae |
| *Antidesma vogelii* | 2 | Phyllanthaceae |
| *Bridelia micrantha* | 7 | Phyllanthaceae |
| *Hymenocardia sp.* | 2 | Phyllanthaceae |
| *Hymenocardia ulmoides* | 2 | Phyllanthaceae |
| *Keayodendron bridelioides* | 4 | Phyllanthaceae |
| *Margaritaria discoidea* | 11 | Phyllanthaceae |
| *Spondianthus preussi* | 1 | Phyllanthaceae |
| *Uapaca guineensis* | 55 | Phyllanthaceae |
| *Uapaca sp.* | 4 | Phyllanthaceae |
| *Oldfieldia sp.* | 1 | Picrodendraceae |
| *Podocarpus latifolius* | 3 | Podocarpaceae |
| *Drypetes aframensis* | 42 | Putranjivaceae |
| *Drypetes aylmeri* | 15 | Putranjivaceae |
| *Drypetes leonensis* | 9 | Putranjivaceae |
| *Drypetes molunduana* | 7 | Putranjivaceae |
| *Drypetes principum* | 45 | Putranjivaceae |
| *Drypetes sp.* | 306 | Putranjivaceae |
| *Lasiodiscus mannii* | 1 | Rhamnaceae |
| *Lasiodiscus sp.* | 1 | Rhamnaceae |
| *Maesopsis eminii* | 4 | Rhamnaceae |
| *Aidia micrantha* | 4 | Rubiaceae |
| *Aulacocalyx sp.* | 2 | Rubiaceae |
| *Brenania brieyi* | 1 | Rubiaceae |
| *Craterispermum sp.* | 3 | Rubiaceae |
| *Dictyandra sp.* | 2 | Rubiaceae |
| *Heinsia crinita* | 1 | Rubiaceae |
| *Massularia acuminata* | 2 | Rubiaceae |
| *Mitragyna sp.* | 1 | Rubiaceae |
| *Morinda lucida* | 1 | Rubiaceae |
| *Nauclea diderrichii* | 6 | Rubiaceae |
| *Nauclea pobeguinii* | 1 | Rubiaceae |
| *Oxyanthus sp.* | 1 | Rubiaceae |
| *Pauridiantha efferata* | 2 | Rubiaceae |
| *Pausinystalia macroceras* | 32 | Rubiaceae |
| *Pausinystalia sp.* | 2 | Rubiaceae |
| *Pausinystalia talbotii* | 1 | Rubiaceae |
| *Pausinystalia yohimbe* | 2 | Rubiaceae |
| *Porterandia cladantha* | 1 | Rubiaceae |
| *Psychotria venosa* | 4 | Rubiaceae |
| *Psydrax sp.* | 1 | Rubiaceae |
| *Rothmannia sp.* | 8 | Rubiaceae |
| *Schumanniophyton magnificum* | 1 | Rubiaceae |
| *Tricalysia sp.* | 22 | Rubiaceae |
| *Zanthoxylum gilletii* | 3 | Rutaceae |
| *Zanthoxylum sp.* | 5 | Rutaceae |
| *Homalium letestui* | 6 | Salicaceae |
| *Oncoba blackii* | 44 | Salicaceae |
| *Oncoba glauca* | 6 | Salicaceae |
| *Oncoba sp.* | 2 | Salicaceae |
| *Oncoba welwitschii* | 6 | Salicaceae |
| *Blighia sp.* | 2 | Sapindaceae |
| *Chytranthus sp.* | 17 | Sapindaceae |
| *Lecaniodiscus cupanioides* | 3 | Sapindaceae |
| *Placodiscus sp.* | 6 | Sapindaceae |
| *Chrysophyllum sp.* | 2 | Sapotaceae |
| *Englerophytum hallei* | 1 | Sapotaceae |
| *Englerophytum sp.* | 4 | Sapotaceae |
| *Pouteria robusta* | 1 | Sapotaceae |
| *Synsepalum msolo* | 3 | Sapotaceae |
| *Tridesmostemon omphalocarpoides* | 4 | Sapotaceae |
| *Brazzea sp.* | 8 | Scytopetalleceae |
| *Quassia sanguinea* | 2 | Simaroubaceae |
| *Quassia sp.* | 1 | Simaroubaceae |
| *Dicranolepis sp.* | 2 | Thymelaeaceae |
| *Liana* | 63 | Undefined |
| *Unknown* | 288 | Undefined |
| *Musanga cecropioides* | 8 | Urticaceae |
| *Rinorea dentata* | 1 | Violaceae |
| *Rinorea oblongifolia* | 71 | Violaceae |
| *Rinorea sp.* | 4 | Violaceae |

**Table S3.** Plant species list (≥10 cm DBH), number of stems per species and family for ecotone-Ganga

| **Species** | **Count** | **Family** |
| --- | --- | --- |
| *Dasylepis racemosa* | 4 | Achariaceae |
| *Lannea acida* | 153 | Anacardiaceae |
| *Pseudospondias microcarpa* | 31 | Anacardiaceae |
| *Pseudospondias sp.* | 3 | Anacardiaceae |
| *Sorindeia sp.* | 134 | Anacardiaceae |
| *Anisophyllea sp.* | 10 | Anisophylleaceae |
| *Duguetia sp.* | 1 | Annonaceae |
| *Duguetia staudtii* | 13 | Annonaceae |
| *Greenwayodendron suaveolens* | 6 | Annonaceae |
| *Monodora myristica* | 1 | Annonaceae |
| *Monodora sp.* | 2 | Annonaceae |
| *Xylopia aethiopica* | 374 | Annonaceae |
| *Xylopia rubescens* | 3 | Annonaceae |
| *Xylopia sp.* | 1 | Annonaceae |
| *Funtumia elastica* | 16 | Apocynaceae |
| *Holarrhena floribunda* | 154 | Apocynaceae |
| *Rauvolfia vomitoria* | 6 | Apocynaceae |
| *Tabernaemontana crassa* | 15 | Apocynaceae |
| *Voacanga poecilocalyx* | 2 | Apocynaceae |
| *Voacanga sp.* | 2 | Apocynaceae |
| *Borassus aethiopum* | 1 | Arecaceae |
| *Fernandoa ferdinandi* | 11 | Bignoniaceae |
| *Fernandoa sp.* | 15 | Bignoniaceae |
| *Markhamia lutea* | 1 | Bignoniaceae |
| *Markhamia sp.* | 5 | Bignoniaceae |
| *Spathodea campanulata* | 1 | Bignoniaceae |
| *Canarium schweinfurthii* | 28 | Burseraceae |
| *Dacryodes sp.* | 16 | Burseraceae |
| *Mammea africana* | 2 | Calophyllaceae |
| *Celtis milbraedii* | 1 | Cannabaceae |
| *Celtis sp.* | 4 | Cannabaceae |
| *Maranthes sp.* | 2 | Chrysobalanaceae |
| *Parinari excelsa* | 23 | Chrysobalanaceae |
| *Garcinia mannii* | 1 | Clusiaceae |
| *Garcinia sp.* | 7 | Clusiaceae |
| *Symphonia globulifera* | 1 | Clusiaceae |
| *Combretum sp.* | 5 | Combretaceae |
| *Terminalia sp.* | 10 | Combretaceae |
| *Terminalia superba* | 6 | Combretaceae |
| *Diospyros sp.* | 32 | Ebenaceae |
| *Bridelia micrantha* | 1 | Euphorbiaceae |
| *Dichostemma glaucescens* | 1 | Euphorbiaceae |
| *Discoglypremna caloneura* | 2 | Euphorbiaceae |
| *Macaranga barteri* | 5 | Euphorbiaceae |
| *Macaranga hurifolia* | 4 | Euphorbiaceae |
| *Macaranga sp.* | 2 | Euphorbiaceae |
| *Maesobotrya barteri* | 1 | Euphorbiaceae |
| *Maesobotrya klaineana* | 2 | Euphorbiaceae |
| *Maesobotrya sp.* | 3 | Euphorbiaceae |
| *Mallotus oppositifolius* | 13 | Euphorbiaceae |
| *Mallotus sp.* | 2 | Euphorbiaceae |
| *Maprounea membranacea* | 2 | Euphorbiaceae |
| *Margaritaria discoidea* | 7 | Euphorbiaceae |
| *Plagiostyles africana* | 1 | Euphorbiaceae |
| *Shirakiopsis elliptica* | 19 | Euphorbiaceae |
| *Tetrorchidium didymostemon* | 2 | Euphorbiaceae |
| *Harungana madagascariensis* | 1 | Hypericaceae |
| *Vismia sp.* | 1 | Hypericaceae |
| *Desbordesia glaucescens* | 4 | Irvingiaceae |
| *Irvingia gabonensis* | 1 | Irvingiaceae |
| *Irvingia grandifolia* | 1 | Irvingiaceae |
| *Irvingia sp.* | 3 | Irvingiaceae |
| *Klainedoxa gabonensis* | 26 | Irvingiaceae |
| *Ochthocosmus sp.* | 21 | Ixonanthaceae |
| *Vitex doniana* | 156 | Lamiaceae |
| *Vitex grandifolia* | 2 | Lamiaceae |
| *Beilschmiedia sp.* | 5 | Lauraceae |
| *Afzelia africana* | 33 | Leguminosae |
| *Albizia adianthifolia* | 4 | Leguminosae |
| *Albizia africana* | 1 | Leguminosae |
| *Albizia glaberrima* | 3 | Leguminosae |
| *Albizia sp.* | 8 | Leguminosae |
| *Amphimas pterocarpoides* | 3 | Leguminosae |
| *Amphimas sp.* | 3 | Leguminosae |
| *Anthonotha ferruginea* | 7 | Leguminosae |
| *Anthonotha macrophylla* | 2 | Leguminosae |
| *Anthonotha sp.* | 8 | Leguminosae |
| *Aubrevillea* | 2 | Leguminosae |
| *Baikiaea sp.* | 1 | Leguminosae |
| *Berlinia bracteosa* | 5 | Leguminosae |
| *Berlinia sp.* | 128 | Leguminosae |
| *Detarium sp.* | 54 | Leguminosae |
| *Dialium dinklagei* | 2 | Leguminosae |
| *Dialium sp.* | 4 | Leguminosae |
| *Dichrostachys cinerea* | 2 | Leguminosae |
| *Erythrophleum suaveolens* | 9 | Leguminosae |
| *Hylodendron gabunense* | 8 | Leguminosae |
| *Parkia bicolor* | 23 | Leguminosae |
| *Parkia biglobosa* | 23 | Leguminosae |
| *Parkia sp.* | 116 | Leguminosae |
| *Piptadeniastrum africanum* | 5 | Leguminosae |
| *Pterocarpus milbraedii* | 1 | Leguminosae |
| *Swartzia fistuloides* | 1 | Leguminosae |
| *Swartzia sp.* | 1 | Leguminosae |
| *Tetrapleura tetraptera* | 29 | Leguminosae |
| *Bombax sp.* | 1 | Malvaceae |
| *Ceiba pentandra* | 10 | Malvaceae |
| *Cola chlamydantha* | 7 | Malvaceae |
| *Cola cordifolia* | 12 | Malvaceae |
| *Cola lateritia* | 6 | Malvaceae |
| *Cola sp.* | 117 | Malvaceae |
| *Desplatsia sp.* | 1 | Malvaceae |
| *Duboscia sp.* | 1 | Malvaceae |
| *Eribroma oblonga* | 3 | Malvaceae |
| *Sterculia sp.* | 2 | Malvaceae |
| *Sterculia tragacantha* | 1 | Malvaceae |
| *Carapa sp.* | 29 | Meliaceae |
| *Guarea sp.* | 1 | Meliaceae |
| *Guarea thompsonii* | 1 | Meliaceae |
| *Khaya grandifolia* | 3 | Meliaceae |
| *Khaya sp.* | 3 | Meliaceae |
| *Lovoa trichilioides* | 35 | Meliaceae |
| *Trichilia prieureana* | 1 | Meliaceae |
| *Trichilia rubescens* | 21 | Meliaceae |
| *Trichilia sp.* | 6 | Meliaceae |
| *Bersama engerana* | 1 | Melianthaceae |
| *Antiaris africana* | 1 | Moraceae |
| *Antiaris sp.* | 1 | Moraceae |
| *Ficus mucuso* | 1 | Moraceae |
| *Ficus sp.* | 9 | Moraceae |
| *Ficus sur* | 2 | Moraceae |
| *Milicia excelsa* | 38 | Moraceae |
| *Treculia africana* | 8 | Moraceae |
| *Treculia sp.* | 2 | Moraceae |
| *Trilepisium madagascariensis* | 15 | Moraceae |
| *Coelocaryon preussii* | 1 | Myristicaceae |
| *Pycnanthus angolensis* | 25 | Myristicaceae |
| *Staudtia kamerunensis* | 4 | Myristicaceae |
| *Syzygium sp.* | 37 | Myrtaceae |
| *Ochna afzelii* | 242 | Ochnaceae |
| *Ochna membrancea* | 2 | Ochnaceae |
| *Ochna sp.* | 58 | Ochnaceae |
| *Heisteria parviflora* | 2 | Olacaceae |
| *Heisteria sp.* | 1 | Olacaceae |
| *Olax subscorpioidea* | 76 | Olacaceae |
| *Strombosia grandifolia* | 4 | Olacaceae |
| *Strombosia sp.* | 4 | Olacaceae |
| *Bartieria fistulosa* | 1 | Passifloraceae |
| *Antidesma membranacea* | 11 | Phyllanthaceae |
| *Antidesma sp.* | 125 | Phyllanthaceae |
| *Drypetes aframensis* | 2 | Phyllanthaceae |
| *Drypetes sp.* | 55 | Phyllanthaceae |
| *Hymenocardia acida* | 27 | Phyllanthaceae |
| *Hymenocardia lyrata* | 573 | Phyllanthaceae |
| *Spondianthus preussii* | 288 | Phyllanthaceae |
| *Uapaca guineensis* | 194 | Phyllanthaceae |
| *Uapaca sp.* | 153 | Phyllanthaceae |
| *Maesopsis eminii* | 2 | Rhamnaceae |
| *Aidia sp.* | 2 | Rubiaceae |
| *Dictyandra involucrata* | 1 | Rubiaceae |
| *Gaertnera longevaginalis* | 4 | Rubiaceae |
| *Gaertnera sp.* | 1 | Rubiaceae |
| *Mitragyna ciliata* | 8 | Rubiaceae |
| *Nauclea pobeguinii* | 1 | Rubiaceae |
| *Nauclea sp.* | 1 | Rubiaceae |
| *Rothmannia sp.* | 1 | Rubiaceae |
| *Tricalysia sp.* | 31 | Rubiaceae |
| *Vepris sp.* | 1 | Rutaceae |
| *Zanthoxylum sp.* | 4 | Rutaceae |
| *Dovyalis zenkeri* | 1 | Salicaceae |
| *Homalium letestui* | 2 | Salicaceae |
| *Homalium sp.* | 2 | Salicaceae |
| *Oncoba dentata* | 3 | Salicaceae |
| *Oncoba sp.* | 12 | Salicaceae |
| *Oncoba spinosus* | 2 | Salicaceae |
| *Oncoba welwitschii* | 24 | Salicaceae |
| *Allophylus africanus* | 9 | Sapindaceae |
| *Blighia sp.* | 1 | Sapindaceae |
| *Chytranthus sp.* | 2 | Sapindaceae |
| *Deinbollia sp.* | 1 | Sapindaceae |
| *Eriocoelum macrocarpum* | 2 | Sapindaceae |
| *Eriocoelum sp.* | 3 | Sapindaceae |
| *Donella sp.* | 1 | Sapotaceae |
| *Englerophytum sp.* | 30 | Sapotaceae |
| *Gambeya boukokoensis* | 9 | Sapotaceae |
| *Gambeya sp.* | 74 | Sapotaceae |
| *Malacantha alnifolia* | 7 | Sapotaceae |
| *Pouteria sp.* | 24 | Sapotaceae |
| *Synsepalum sp.* | 72 | Sapotaceae |
| *Quassia sanguinea* | 6 | Simaroubaceae |
| *Quassia sp.* | 7 | Simaroubaceae |
| *Liana* | 170 | Undefined |
| *Unknown* | 235 | Undefined |
| *Myrianthus arboreus* | 44 | Urticaceae |
| *Rinorea oblongifolia* | 7 | Violaceae |

**Table S4.** Twenty most important chimpanzee plant food species consumed per site.

| Bekob | | | Njuma | | | Ganga | | |
| --- | --- | --- | --- | --- | --- | --- | --- | --- |
| Species | Stems | BA | Species | Stems | BA | Species | Stem | BA |
| *Antiaris toxicaria* ^1^ | 2 | 0.2134 | *Annickia chloranta* | 23 | 1.6016 | *Antidesma* sp. | 125 | 3.4566 |
| Antidesma sp. | 71 | 1.7413 | *Antiaris toxicaria* ^1^ | 2 | 1.7843 | ***Canarium schweinfurthii*** | 28 | 6.2835 |
| ***Antrocaryon klaineanum*** ^2^ | 3 | 0.7658 | *Antidesma* sp. | 27 | 0.6211 | *Drypetes* sp. | 56 | 1.5653 |
| *Canarium schweinfurthii* | 2 | 1.1082 | *Canarium schweinfurthii* | 1 | 0.2686 | *Duguetia* sp. | 13 | 0.5207 |
| ***Cleistopholis patens*** | 6 | 2.2584 | *Cleistopholis patens* | 6 | 0.2671 | *Ficus* spp. ^2^ | 12 | 4.5825 |
| *Elaeis guineensis* ^2^ | 33 | 3.6423 | *Duboscia macrocarpa* | 3 | 1.2431 | *Irvingia gabonensis* ^1^ | 5 | 0.1074 |
| *Ficus* spp. ^2^ | 12 | 3.0736 | *Ficus* spp. ^2^ | 8 | 1.5668 | ***Landolphia*** sp. | 170 | 2.3282 |
| *Grewia coriacea* | 47 | 2.8972 | ***Grewia coriacea*** | 34 | 4.2324 | *Lannea welwitschii* | 153 | 5.8903 |
| ***Landolphia* sp.** ^2^ | 70 | 1.2234 | ***Landolphia*** sp. | 63 | 0.9936 | *Milicia excelsa* ^2^ | 38 | 7.2739 |
| *Mammea africana* | 25 | 0.6662 | *Musanga cecropioides* ^2^ | 8 | 0.5358 | *Monodora myristica* | 3 | 0.1439 |
| *Monodora myristica* ^1^ | 31 | 1.1018 | *Nuclea diderrichii* ^2^ | 6 | 2.6566 | ***Myrianthus arboreus*** | 44 | 2.4211 |
| ***Musanga cecropioides*** ^2^ | 9 | 0.5961 | *Pseudospondias microcarpa* ^2^ | 2 | 0.0725 | *Olax subscorpioidea* ^2^ | 76 | 1.3783 |
| *Nuclea diderrichii* ^2^ | 4 | 0.733 | ***Pycnanthus angolensis*** ^2^ | 87 | 47.2509 | ***Pseudospondias microcarpa*** | 34 | 9.4913 |
| *Pseudospondias microcarpa* ^2^ | 28 | 3.2708 | *Ricinodendron heudelotii* ^1^ | 5 | 5.7359 | *Pycnanthus angolensis* | 25 | 3.3597 |
| ***Pycnanthus angolensis*** | 112 | 28.8224 | *Santiria trimera* | 43 | 5.7939 | *Staudtia kamerunensis* | 4 | 0.221 |
| *Ricinodendron heudelotii* | 3 | 2.4855 | *Staudtia kamerunensis* | 57 | 7.691 | ***Synsepalum*** sp. | 72 | 2.9902 |
| ***Santiria trimera*** | 84 | 18.9064 | *Tabernaemontana crassa* | 110 | 2.6796 | ***Uapaca guineensis*** ^2^ | 194 | 12.0802 |
| *Trichoscypha* sp. | 92 | 4.8077 | *Treculia* sp. | 22 | 0.6056 | *Uapaca togoensis* | 153 | 11.8478 |
| ***Uapaca guineensis*** ^2^ | 155 | 11.3381 | *Trichoscypha* sp. | 28 | 2.4177 | *Vitex doniana* | 156 | 10.7761 |
| *Uapaca* sp. | 10 | 0.9404 | ***Uapaca guineensis*** ^2^ | 55 | 7.956 | *Vitex grandifolia* | 2 | 1.2538 |

^1^ Dry season fruiting species, ^2^ asynchronous (dry and wet season) fruiting species, and bold: species present in ≥50% of monthly samples and/or accounting for ≥50% of monthly fruit consumption volume for site
